# Mechanistic Insights and Clinical Implications of ELK1 in Solid Tumors: A Narrative Review

**DOI:** 10.3390/cells14161257

**Published:** 2025-08-14

**Authors:** Georgios Kalampounias, Theodosia Androutsopoulou, Panagiotis Katsoris

**Affiliations:** 1 Laboratory of Cell Biology, Division of Genetics, Cell and Developmental Biology, Department of Biology, School of Natural Sciences, University of Patras, 26504 Patras, Greece; tandroutsopoulou@ac.upatras.gr; 2 Institute for Bioinnovation, Biomedical Sciences Research Centre “Alexander Fleming”, 16672 Athens, Greece

**Keywords:** MAPKs, ETS family, SRF, SRE, glioblastoma, tumorigenesis, invasion, immediate early genes, oncogene, epithelial-to-mesenchymal transition

## Abstract

ELK1 is a Transcription factor (TF) belonging to the ETS-domain TF family, mainly activated via RAS-RAF-MEK-ERK signaling. As a nethermost pathway molecule, ELK1 binds to Serum-response elements (SREs) and directly regulates the transcription of Immediate early genes (IEGs) including *FOS* and *EGR1*. Due to ELK1’s influence on key cellular processes such as proliferation, migration, apoptosis evasion, and Epithelial-to-mesenchymal transition (EMT), its role as a key contributor to tumorigenesis is emerging. In recent years, elevated expression and/or activation of ELK1 has been reported in various malignancies, including lung, breast, prostate, colorectal, blood, gastric, liver, cervical, thyroid and ovarian cancer. ELK1 acts primarily through direct DNA binding but also through interaction with other oncogenes, noncoding RNA molecules, TFs, and upstream kinases (other than ERK1/2), thus participating in diverse axes of transcriptional regulation. Its crucial role in IEG expression has been particularly implicated in cancer progression, metastasis, and drug resistance. Owing to its role in multiple cellular functions and its subsequent oncogenic potential, further elucidation of intracellular ELK1 interactions is of paramount importance. This review aims to summarize current evidence on ELK1’s involvement in solid tumors, dissect reported mechanistic roles, and highlight recent insights that could fuel future ventures of high translational interest.

## 1. Introduction

Cancer is a multiparametric disease affecting millions of people, being responsible for millions of deaths throughout human history. The term per se does not describe a unique disease or condition but rather a set of diseases that affect different cell types, tissues, and organs and are grouped together by a set of common hallmarks [1]. It is noteworthy that although each cancer case is unique and the disease is highly dependent on several genetic and environmental factors, the mechanisms underlying the emergence and development of the tumor, namely tumorigenesis, often converge and over the years, molecules of high significance have been identified and targeted. A common characteristic of all cancer types is the ability of the malignant cells to proliferate, defying the cytostatic signals from the various homeostatic mechanisms (both intracellular and extracellular) [2]. This function is mediated by oncogenes, the expression of which is dysregulated in tumors as they uncontrollably promote cell cycle progression, metabolic changes to sustain mitosis, and ultimately lead to increased proliferation rate [1,2]. The activity of oncogenes in normal cells is suppressed at multiple levels, including methylation, histone modifications, decreased transcription, rapid degradation of the gene product, and post-translational modifications that affect the proteins’ turnover time [2,3,4]. However, in cancer cells, these functions gradually shift from the inactive to the active mode, mainly through the expression of proteinic effectors that undertake such changes [5,6]. In this tumorigenic process, transcription factors (TFs) are huge contributors since they control the expression of several downstream genes and serve as hubs of signal integration [5,6].

The E26 transformation-specific (ETS) family is a group of 28 TFs, sharing homologous sequences, which have been found to regulate several processes, many of which have been associated with development and diseases [7,8,9]. All ETS TFs share a highly conserved DNA binding domain, namely the ETS domain, which a winged helix-turn-helix (HLH) motif that binds DNA regions high in purines and a central GGA sequence [10]. Besides this sequence, they also interact with other proteins and TFs, forming complexes and jointly regulating the expression of genes upon binding to the respective promoters [11,12]. Depending on their phosphorylation status, ETS TFs can act as transcriptional repressors or activators of their target genes and have been found to regulate cellular differentiation, cell cycle progression, proliferation, migration, angiogenesis, and even apoptosis, thus participating in multiple functions [13,14,15]. Due to their participation in core cellular functions, most of them have been reported in the context of cancer, with many roles correlated to their expression and activation [16]. Although several studies about the role of ETS TFs have been conducted, not all ETS family members have been studied thoroughly throughout the years. ELK1 is one such member, a protein which acts mainly as an ERK1/2 effector and acts as an integration point for several upstream and downstream signals.

ELK1 has been studied in the context of memory, addiction, and neurogenerative diseases [9,17,18]; however, studies implicating it in tumor-associated process are not rare [19,20,21]. In fact, over the last three decades it has been reported in hundreds of papers, mostly implicated in tumorigenesis (both of solid tumors and hematological malignancies), cancer progression, drug resistance and often credited with significant diagnostic/prognostic value. Hence, the purpose of this study is to focus on ELK1, a TF which has repeatedly been reported in numerous cancer-associated phenomena, and review existing literature regarding the protein’s role. The ever-emerging fields of micro-RNA biology and drug-resistance research advocate for more research on ELK1 since it is a protein both regulating the expression of several micro-RNAs and being implicated in chemoresistance in advanced disease stages.

## 2. ELK1 in Solid Tumors and Metastatic Cells

### 2.1. Lung Cancer

In recent years, several studies have been conducted in lung cancer concerning ELK1’s role in tumorigenesis and tumor progression. A 2022 study by Lie et al. identified ELK1 as the TF mediating B cell lymphoma 6 (BCL6) gene expression following stimulation from the RAS-RAF-MEK-MAPK pathway [22]. Due to the prevalence of *KRAS* mutations, MAPK signaling is constantly active, promoting the promotion of antiapoptotic proteins like BCL6, explaining BCL6’s overexpression detected in lung tumors [22]. BCL6 inhibition was found to KRAS-activated pro-survival signal transduction, both in vivo and in vitro, elucidating how a ELK1-dependent pathway can lead to apoptosis resistance [22]. In KRAS-mutated lung adenocarcinoma (LUAD) (having the KRAS^G12C^ mutation), the T-LAK cell-originated protein kinase (TOPK) has been found significantly upregulated [23]. TOPK is a well-established serine/threonine MAPK-like protein contributing to tumor progression [24]. Cai et al. in 2023 demonstrated that ELK1 is the TF regulating TOPK expression, thus providing the connecting link between KRAS and TOPK expression [23]. Another study investigated the relation between ELK1 and autophagy regulation and reported that under heat stress conditions, Heat shock protein 27 (HSP27) can promote ELK1’s activation, leading to the activation of autophagy [25]. Regarding ELK1’s role in tumor promotion, besides its activation via the RAS-RAF-MEK-MAPK pathway, significant insight is now available about the epigenetic regulation of its expression. In LUAD, overexpression of Heterochromatin protein 1γ (HP1γ, also called CBX3) has been reported, which has been shown to amplify KRAS-driven signaling, while its silencing leads to less aggressive tumors [26]. HP1γ was found to act by downregulating the expression of the transcription-repressive regulators Nuclear receptor corepressor 2 (NCOR2) and Zinc finger and BTB domain containing 7A (ZBTB7A), which upon expression significantly downregulate ELK1 and AXL receptor tyrosine kinase (AXL) [26]. This mechanism highlighted how epigenetic modification can activate ELK1-mediated signaling and form positive loops, since ELK1 has been reported to participate in the epigenetic activation of other oncogenes by other researchers [26].

ELK1 is often implicated in the regulation of other TFs, including their activation by forming complexes, their transcription, or the silencing of their inhibitors. In KRAS-mutated NSCLC, a 2012 study reported elevated levels of Mediator of RNA polymerase II transcription subunit 23 (MED23) [27]. MED23 was found to promote cell proliferation and growth, and its silencing led to suppression of RAS-related pro-oncogenic activity [27]. ELK1 was found to be a significant part of this connection since it is the most downstream component of the RAS-RAF-MEK-MAPK-ELK1 pathway and acts by co-regulating oncogenes with MED23 [27]. The expression of MED23 is found upregulated following Ras stimulation and follows the activation pattern of ELK1 [27]. It was also identified as a potential pharmaceutical target and as a prognostic factor, since patients with a lower MED23 expression had better survival [27].

ELK1 has been reported to be a significant promoter of Epithelial to mesenchymal transition (EMT) in NSCLC and especially LUAD [28]. A 2021 study states that ELK1 promotes the transcription of B7 homolog 3 (B7-H3 or CD276), a transmembrane protein with immunoregulatory activity [28]. ELK1 was reported to be overexpressed in LUAD, and its role as a poor prognosis factor was highlighted [28]. The study demonstrated that ELK1 binds to the promoter of the *B7H3* gene and actively promotes its transcription [28]. Regarding metastasis and vascular invasion, ELK1 was found to be the downstream target of Transforming growth factor beta 1 (TGF-β1) stimulation, leading to the upregulation of Pleckstrin 2 (PLEK2) in NSCLC cells [29]. Both ELK1 and PLEK2 were found to be overexpressed in lung cancer, and prognostic value was also attributed to their expression level. PLEK2 was found to be a driver of EMT and metastasis promotion, and PLEK2-knockdown cells were shown to be insensitive to TGF-β1 pro-EMT activity [29]. Another pathway was described by Khan et al. in 2016 [30]. Activated ELK1 (via KRAS oncogenic signaling) in LUAD cells was documented to form a complex with E1A binding protein/Histone acetyltransferase p300 (commonly known as p300), induce the p65 (RelA or NF-κB p65) DNA binding, and promote the transcription of Snail family transcriptional repressor 2 (Slug or SNAI2) [30]. Slug is a transcriptional repressor of E-cadherin and thus facilitates cadherin switch [31]. This pathway was identified to be a strong EMT driver and was effectively inhibited by aspirin by inhibiting the nuclear translocation of p65 and reversing the complex’s pro-EMT activity [30]. Interactions of lung cancer cells with other cell types have also been shown to affect intracellular signaling, including the expression and activation of ELK1. A 2015 study investigated the crosstalk between NSCLC cells and stromal fibroblasts in vitro, aiming to characterize EMT signatures [32]. ELK1 was among the TFs reported as upregulated, with the others being NF-κβ, Activator Protein 1 (AP-1), Hypoxia-inducible factor 1-alpha (HIF-1A), Krüppel-like factor 4 (KLF4; also known as Epithelial Zinc Finger protein, EZF), and Specificity protein 1 (SP1) [32]. Another study focused on the interactions of NSCLC cells with elements of the immune system [33]. Peripheral blood mononuclear cells (PBMCs) were examined, and what was found was that in the presence of a lung tumor, the expression of ELK1, ELK4, and SP1 is elevated, emphasizing a potential role of PBMCs’ ELK1 levels as a diagnostic tool [33]. The study also reports that following tumor removal, the expression of more than 3000 (previously) upregulated/downregulated genes returns to baseline levels, further supporting PBMCs as a non-invasive diagnostic tool [33].

Moreover, ELK1 has been mentioned several times regarding its role in the regulation of noncoding RNAs (Table 1). A 2018 study by Sheng et al., reported that n LUAD, ELK1 can upregulate the transcription of the lncRNA HOXA10-AS, which may be an RNA of increased prognostic value [34]. The study reports that HOXA10-AS silencing leads to decreased proliferation, migration, and EMT progression, while the apoptotic activity of HOXA10-AS-knockdown cells is increased [34]. LncRNA HOXA10-AS was found to act by positively regulating Wnt/β-catenin signaling and ELK1 overexpression was reported to further amplify this phenomenon [34]. ELK1 has also been found to facilitate miR-30c and miR-21 transcriptional activation following stimulation from KRAS oncogenic signaling [35]. A 2018 study by Shi et al. demonstrated that the micro-RNAs miR-30c and miR-21 are significantly overexpressed in KRAS overexpressing tumors (both wild type and KRAS^G12D^ mutants) and participate in drug resistance, cell migration and invasion, through the inhibition of tumor suppressor genes such as Neurofibromin 1 (*NF1*), Ras GTPase-activating protein 1 (*RASA1*), BH3 interacting domain death agonist (*BID*), and Ras association domain-containing protein 8 (*RASSF8*) [35]. Another mechanism involving ELK1 in noncoding RNA transcription regulation is the case of miR-34a [36]. miR-34a has been found to participate in a negative feedback loop that regulates AXL expression levels [36]. Cho et al. in 2016 reported that AXL overexpression activated c-Jun N-terminal kinases (JNKs) signaling which phosphorylates ELK1, the TF facilitating the transcription of miR-34a [36]. miR-34a then targets the *AXL* mRNA and reduces its translation, thus limiting AXL synthesis and forming a regulating mechanism that can regulate chemoresistance and apoptosis [36].

**Table 1:** ELK1 targeting long noncoding RNAs (lncRNAs) in cancer.

Cancer	lncRNA	Regulation by ELK1	Mechanism of Action	Outcome	Ref.
NSCLC	lncRNA HOXA10-AS	Upregulation	Positive regulation of Wnt/β-catenin signaling	Promotion of proliferation, migration, and EMT progression	[34]
CRC	lncRNA LBX2-AS1	Upregulation	Blocking of the degradation of S100 calcium binding protein A11 (S100A11 or MLN70) Targets the tumor suppressor miR-491-5p	Promotion of proliferation, migration, and invasion	[37]
GC	lncRNA TRPM2-AS	Upregulation	Targets the tumor suppressor miR-195	Promotion of invasion and increases in metastatic potential	[38]
GC	lncRNA MIR100HG	Upregulation	Positive regulation of TGF-β, Wnt/β-catenin, Hippo, and MAPK signaling	Promotion of proliferation, migration, and invasion	[39]
PTC	lncRNA LINC01638	Upregulation	Positive regulation of intracellular signaling cascades leading to c-MYC activation	Promotion of cell cycle progression, proliferation, migration, and invasion	[40]
OC	lncRNA LBX2-AS1	Upregulation	Targets the regulatory micro-RNA miR-4784	Promotion of cancer progression	[41]
Glioma	lncRNA PSMB8-AS1	Upregulation	lncRNA PSMB8-AS1 downregulates the expression of miR-574-5p	Promotion of proliferation	[42]
Osteosarcoma	lncRNA MIR100HG	Upregulation	Positive regulation of TGF-β, Wnt β-catenin, Hippo, and MAPK signaling	Promotion of cancer progression	[43]
Osteosarcoma	lncRNA LINC02381	Upregulation	Targets the regulatory micro-RNA miR-503-5p	Promotion of proliferation	[44]

Abbreviations: NSCLC = Non-small cell lung cancer; CRC = Colorectal cancer; GC = Gastric cancer; PTC = Papillary thyroid carcinoma; OC = Ovarian cancer; EMT = Endothelial to mesenchymal transition.

Targeting ELK1’s activation has been reported to be a cancer-preventative strategy. Carvedilol, a known β-blocker, has been reported to be implicated in anti-tumorigenic mechanisms. In human bronchial epithelial cells, carvedilol was found to inhibit the carcinogenic activity of benzo(a)pyrene diol epoxide (BPDE), which acts by activating ELK1 and drives lung tumorigenesis [45]. In another approach, Hexagonal selenium nanoparticles modified by siRNA (HSNM-siRNA) were administered to NSCLC cells to inhibit Epidermal Growth Factor Receptor (EGFR/HER1/ErbB1) signaling and the intracellular accumulation of several TFs (NF-κΒ, c-MYC, STATs, and ELK1) was assessed [46]. All TFs were found to be downregulated, upon exposure to the HSNM-siRNA and cell cycle arrest, reduced viability, and apoptosis induction were documented [46]. Another study investigated the effects of Insulin-like growth factor binding protein-3 (IGFBP-3), which has been reported to suppress cell proliferation, migration, invasion, and angiogenesis by both IGF-dependent and IGF-independent pathways [47]. Using NSCLC cells as well as head and neck squamous cell carcinoma, this 2011 study showed that IGFBP-3 leads to decreased ERK1/2 phosphorylation, by direct interaction with the MAPKs (in a non-IGF-dependent manner), thus limiting ELK1’s transcriptional activity [47]. In NSCLC, another poor prognosis factor has been found to be the downregulation of Ephrin type-B receptor 6 (EPHB6) [48]; a Receptor tyrosine kinase (RTK), the loss of which promotes metastasis. Cells engineered to overexpress a kinase-defective EPHB6 receptor, upregulated MAPK signaling; nonetheless the authors reported that this did not lead to ELK1 activation [48]. Even though the study did not elaborate further, cases of ERK1/2 activation without ELK1 phosphorylation have been reported elsewhere and seem to be a result of ERK1/2 translocation-inhibition. Increases in the cytoplasmic fraction of ERK1/2 do not always lead to ELK1’s activation. Therefore, the phenomenon studied by Yu et al. in 2009 could be a result of such a mechanism [48], implying a potential role of importins in the observed effect. The cytotoxicity of afatinib was reported to be ELK1-dependent according to Chao et al. in 2015 [49]. The study reports that in NSCLC cell lines, afatinib decrease the phosphorylation of ELK1 and this led to a reduction in Cancerous Inhibitor of Protein Phosphatase 2A (CIP2A) gene transcription [49]. Interactions between ELK1 and CIP2A have been reported elsewhere as well, thus accrediting this mechanism with a pan-cancer significance [50,51,52,53]. CIP2A downregulation is reported to promote the activity of Protein Phosphatase 2A (PP2A) activity and thus suppress Protein kinase B (PKB or AKT) phosphorylation [49].

Finally, ELK1 has also been implicated in drug resistance. A 2024 about acquired gefitinib-resistance reports that ribosomal protein S6 kinase 1 (S6K1) and Mammalian Target of rapamycin (mTOR) were found to contribute in gefitinib resistance, both of them being overexpressed and activated [54]. ELK1 was found to directly interact with the mTOR and S6K1 promoters and drive mTOR and S6K1 expression [54]. Moreover, ELK1-kockdown cells had decreased mTOR and S6K1 protein levels and the efficacy of gefitinib was similar to those of non-resistant cells, underscoring the TF’s role in the gefitinib-resistant phenotype [54]. Another study on gefitinib further reinforced ELK1’s role in chemoresistance [55]. In this 2016 study by Duan et al., Peptidylarginine deiminase IV (PAD4) overexpression was found to halt EMT progression and suppress gefitinib resistance in NSCLC cells [55]. PAD4 was reported to act by inhibiting the expression of ELK1 which has an undoubted role in EMT progression [55]. Besides gefitinib, ELK1 has also been reported in erlotinib resistance [56]. A study conducted on erlotinib-resistant rodent models (having the human EGFR T790M erlotinib-resistance mutation) showed that several transcriptome changes occur that allow for metabolic adaptation, upregulation of oxidative stress defense mechanisms, and tumor progression pathways [56]. The model of the study remained susceptible to a combined scheme of afatinib (BIBW 2992)/rapamycin and the authors concluded that the upregulated pathways were controlled by ELK1, Nuclear respiratory factor 1 (NRF1), Nuclear respiratory factor 2 (NRF2), c-MYC, and Sterol regulatory element-binding protein (SREBP), while also relying heavily on mTORC1-mediated transcription regulation [56].

### 2.2. Breast Cancer

The vast majority on studies conducted on ELK1’s implications in cancer are about Breast cancer (BC). By being one of the most common malignancies (the most frequent affecting women), there is always interest in the identification of new molecules of high diagnostic, prognostic, and therapeutic importance. A 2024 study reported that the expression of ELK1 is higher in BC tissues compared to their normal counterparts and demonstrated that ELK1 silencing leads to BC suppression [57]. Additionally, they credited ELK1 with roles regarding tumor microenvironment (TME) and evasion from the immune system, while also underscoring its high prognostic value [57]. A 2013 study conducted a correlation of ELK1 expression and localization to key BC markers like ER, Cyclin D1, and Ki67, and concluded that ELK1 had a positive correlation with the first two highlighting its significance as a potential marker or prognosis predictor [58]. Bioinformatics analyses have also underscored the significance of ELK1 as an important transcription regulator of BC. A 2008 study based on transcriptomics recognized that motifs associated with poor prognosis in BC are bound by the TFs ELK1, E2F transcription factors, NRF1 and Nuclear Factor (NFY), underscoring the pivotal role of ELK1 in pro-tumorigenic gene transcription [59]. Another study based on computational biology revealed that ELK1 is implicated on c-Fos expression, by altering the epigenome [60]. ELK1 was shown to interact with PAD4, which was correlated to actively transcribed genes in BC and has also been reported in NSCLC [55]. The ELK1-PAD4 interaction was also shown to affect the expression of the *FOS* gene, thus forming a positive feedback loop since *FOS* is a target of EGF signaling cascade, which also activates MAPKs and ELK1 [60].

ELK1 has been reported multiple times to interact with other TFs, including Myeloid Zinc Finger 1 (MZF1) and Tumor protein p53 (TP53). In Triple-negative breast cancer (TNBC), MZF1 has been reported to have a pro-metastatic role [61]. A 2019 study revealed that MZF1’s stability is enhanced by its interaction with ELK1 and both TFs participate in IGF1R-associated signaling [61]. MZF1 was found more potent in the maintenance of EMT-characteristics; however, the loss of ELK1 made the cells susceptible to p38/Slug activation [61]. Later studies correlated the expression of Protein kinase C alpha (PKCα), ELK1 and MZF1 [12,62], and reported them to be higher in BC cell lines of a higher metastatic potential [62]. Moreover, Lee et al. in 2026 showed that impairing the interaction between ELK1 and MZF1 downregulated PKCα expression and suppresses cellular functions like migration, invasion, and EMT, thus emphasizing ELK1’s role in MZF1-ascoaited activity [12]. A recent study revealed that ELK1 also interacts with mutant *TP53* alleles in aggressive forms of BC, to facilitate metastasis by activating the FOS family TF, FOSL1 gene [63].

Extensive studies have been conducted on dysregulated MAPK signaling, and how its activation and ELK1-mediated gene transcription drives BC tumorigenesis, progression, and metastasis. A 2012 study focused on Muscle RAS oncogene homolog (M-Ras/MRAS) and revealed that although Raf-MEK-ERK activation is lower compared to other Ras forms, it can induce the activation of ELK1 both in ERK-dependent and -independent manners [64]. The ERK1/2 activation of ELK1 is well-established; nonetheless, Castro et al. described that M-Ras interacts with RAS-like proto-oncogenes A/B (RALs) leading to the activation of the Ral/JNK pathway and thus supporting the JNK-mediated phosphorylation of ELK1 via this alternative activating route [64]. Another study investigated how RAS-RAF-MEK-ERK activation can lead to the activation of Enhancer of Zeste Homolog 2 (EZH2). The study revealed that the transcription of EZH2 is ELK1-dependent, and that EZH2’s overexpression is associated with aggressive cancer types like TNBC and EGFR-overexpressing BC [65]. This finding has also been reported in other cancer types, in which ELK1 was reported to affect gene expression through the expression of the histone modifier EZH2 [66].

ELK1 has also been found to regulate cytoskeleton-associated phenomena in BC [67]. In a 2023 study, ELK1 was found to control the expression of Kinesin family member C1 (KIFC1), which has been credited with a pivotal role in cell proliferation and oxidative stress regulation via regulation of glutathione (GSH) levels [67]. In another study (2021) ELK1 was recognized as the TF controlling the expression of another kinesin superfamily member, namely Kinesin family member 26B (KIF26B), which has also been implicated in cell cycle progression and proliferation [68]. ELK1 silencing was found to downregulate the expression of both KIFC1 and KIF26B, causing detrimental effects on BC cell proliferation and metastatic potential [67,68].

Several studies on BC have focused on the interaction between ELK1 and growth-factor-initiated signaling. In basal-like breast cancer, a 2020 study identified associations between key receptors such as Tumor necrosis factor receptors (TNFRs), Transforming growth factor-beta receptor type 1 (TGFBR1), and EGFR, and ELK1 [69]. The correlation between ELK1 activation and growth factors is one of the earliest regarding ELK1’s role in cancer. A 1997 study documented the activating effects of Insulin-like growth factor I (IGF-I) and EGF on ERK1/2, lead to the activation of ELK1 [70]. EGF was later found to control the expression of the anti-apoptotic Myeloid cell leukemia sequence 1 protein (MCL1), the transcription of which is regulated by ELK1 [71]. ELK1 was found to be crucial in successful EGFR signaling, and the study also reported a positive correlation of ELK1’s and MCL1’s expression levels in BC tumors, further reinforcing the TF’s role in tumor progression and aggressiveness [71]. ELK1’s activation by EGFR was shown to control the levels of CIP2A, which is a marker of poor prognosis and potential pharmaceutical target [72]. The study focused on the effects of TD52 (an erlotinib derivative with minimal p-EGFR inhibition but significant CIP2A downregulation) in TNBC and concluded that TD52 impaired ELK1’s binding to the CIP2A promoter, thus halting the protein’s expression [72]. In a 2016 study, lapatinib’s mechanism of action was described, including the inhibition of CIP2A/PP2A [73]. Lapatinib was found to decrease ELK1’s phosphorylation by targeting upstream receptors (HER2, EGFR), resulting in a downregulation of the oncogene CIP2A and thus, increasing the expression of its target PP2A [73]. Targeting ELK1-mediated transcription of CIP2A seems to have a pan-cancer significance, since it has been documented in several cancer types [49,50,51,52,53]. Hence, the importance of targeting this mechanism to suppress proliferation and induce apoptosis may be a feasible approach. Another study confirmed the correlation between MAPK/ELK1/CIP2A/PP2A and explored whether targeting of SET nuclear proto-oncogene protein (or simply SET), another PP2A inhibitor can disrupt the oncogenic activity of CIP2A [74]. The authors reported that SET overexpression also led to increased expression of AKT, ERK, ELK1, and CIP2A, indicating a positive feedback loop [74]. Moreover, they suggested the use of TD19 to downregulate *CIP2A* mRNA levels, by decreasing ELK1-mediated transcription of the *CIP2A* gene [74]. Finally, the EGRF/ELK1 axis was found to regulate the expression of Plasminogen activator inhibitor-1 (PAI-1), which has a pivotal role in BC progression and metastasis [75]. ELK1 has also been found to participate in the Cannabinoid receptor 2 (CB2) and HER2 interplay. According to a 2015 study, HER2 upregulated the expression of CB2 by activating ELK1-mediated transcription of CB2, while CB2 activation further stimulates the HER2 pathway by activating c-Src [76]. Therefore, both receptors seem to be components of a positive feedback loop, and the connecting link is ELK1 who regulated the transcription of CB2 [76].

Significant crosstalk between ELK1 and steroid hormone (estrogens, progestins, androgens) interplay has also been reported. Associations between 17beta-estradiol (E2) (alone or in combination with other growth signals) and ELK1 have been reported to regulate the expression of genes involved in several pro-tumorigenic functions. In a 2002 study, it is reported that E2 induces the expression of the c-Fos oncogene via the Estrogen receptor alpha (ERα). ERα was reported to phosphorylate the MAPKs which in turn activate ELK1 and leads to *FOS* transcription [77]. A 2004 study reported that E2 activates MAPK-related signaling and can lead to the activation of EGR1 via ELK1-mediated transcription [78]. Thus, a mechanism of non-genomic E2 actions was described, emphasizing the role of ELK1, and this mechanism was being repeatedly reported by other studies as well [77,78,79]. A more detailed explanation was provided by Santen et al. in 2004 in which estradiol is reported to bind to cell membrane-associated estrogen receptors (ERs) and initiate a cytoplasmic signaling cascade [79]. ERs interact with the Src homology 2 domain-containing adaptor proteins (SHC) and induce their phosphorylation [79]. The authors report that activated SHC binds Growth factor receptor bound protein 2 (GRB2) and Son of sevenless homologs 1 and 2 (SOS1/SOS2), and this cascade leads to activation of the MAPKs, thus explaining ELK1 is ultimately activated [79]. Santen et al. reported that this mechanism may by a significant part of adaptive hypersensitivity and proposed targeting the MAPK pathway as an alternative target adaptive hypersensitivity. Therefore, the role of ELK1 as a mediator of adaptive hypersensitivity to estrogen was underscored. A similar study emphasized how adaptive hypersensitivity to estrogen (resulting from estradiol deprivation), exploit this non-genomic mechanism, upregulate the corresponding genes and thus become dependent on growth factor signaling, mainly though IGF-I receptor (IGF-1R) [80]. A 2005 study reported that prolactin (PRL) and E2 induce the activation of AP-1, by promoting the expression and activation of c-Fos [81]. Both hormones are reported to synergically activate MAPKs (ERK1/2 and p38), leading to ELK1 phosphorylation and the subsequent transcription of FOS gene [81]. A 2010 study reported that E2 and carbachol treatment can induce the proliferation of MCF-7 cells [82]. The phenomenon was found to be Calcium/Calmodulin-dependent kinase (CaM) and the signal cascade was also shown to included ERK1/2 activation and the subsequent phosphorylation of ELK1 [82]. Another study, conducted in 2015 reported E2 and the selective G protein-coupled estrogen receptor (GPER) ligand G-1 promote the expression if miR-144 [83]. miR-144 has been credited with multiple activities in cancer (both pro- and anti-oncogenic, depending on the cancer type), and its expression in SK-BR-3 cells was found to be regulated through GPER and the PI3K-MAPK/ELK1 axis [83]. ELK1 assists the transcription of miR-144 which in turns suppresses the expression of the onco-suppressor Runt-related transcription factor 1 (RUNX1) [83]. Therefore, another example of a non-genomic E2 action was described, in which ELK1 is also an important component. ELK1 has also been reported to participate in a complex transcriptional network with Androgen receptor (AR) [84]. In ER-negative BC, AR was found to promote ERK1/2 activation and lead to activation of ELK1, Ribosomal S6 Kinase 1 (RSK1), and c-Fos [84]. On the other hand, AR downregulation was found to have the opposite effects and decrease ELK1 phosphorylation [84]. Chia et al. also reported that AR-stimulation required HER2 expression in order to lead to its non-genomic effects, while ERK1/2 was also found to regulate AR expression via the activity of cAMP response element binding protein 1 (CREB1), thus forming a complete positive feedback loop that connects AR-MAPK-ELK1 in ER-negative/HER2-positive BC [84].

ELK1 has also been found participating in transcriptional regulation loops with noncoding RNA molecules. A 2013 study reported that ELK1, as well as ETS variant transcription factor 4 (ETV4 or PEA3), regulates the expression of the tumor-suppressing microRNA miR-200b [85]. According to Zhang et al. ELK1 suppresses the expression of miR-200b while miR-200b, upon activation regulates the activity of both ETV4 and ELK1 through regulation of Peptidyl-prolyl cis-trans isomerase NIMA-interacting 1 (PIN1) and thus, the PIN1-ERK1/2 pathway [85]. The expression of ELK1 (and ELK3) has been reported to be the target of miR-135a [86]. A 2018 study by Ahmad et al., showed that miR-135a overexpression reduces ELK1/3 levels, and significantly affects the proliferation of MCF-7 and T47D cells [86]. In a 2020 study, Zhao et al. showed that the circular RNA hsa_circRPPH1_015 is upregulated in BC tissues (in a sample of 86 tissues) and its activity is positively correlated to BC cell proliferation and aggressiveness [87]. The circular RNA hsa_circRPPH1_015 was found to act by sponging miR-326, which is a known suppressor of ELK1 expression in cervical cancer as well [87,88]. Another study further reinforced the correlation between miR-326 and ELK1 in BC [89]. Recently, Wang et al. conclfuded that hsa_circRNA_000166 also acts as on oncogene by sponging both miR-326 and miR-330-5p. Given the fact that a similar mechanism, and both micro-RNAs, have been has been recorded in cervical cancer as well [88,90], hsa_circRPPH1_015 and ssa_circRNA_000166 were recognized as oncogenes, facilitating the dysregulation of ELK1 and leading to more aggressive phenotypes [87,89].

An older study by Maniccia et al., in 2009, reported that the expression and mitochondrial localization of the BRCA1, BRCA1a, and BRCA1b tumor-suppressor genes can inhibit ELK1-mediated signaling, and thus reduce the pro-proliferation activity exhibited by ELK1’s target genes [91]. Therefore, ELK1 was correlated to mitochondrial dysfunctions, which is a hallmark of cancer, and identified a connecting link between ELK1-mediated transcription and the activity of Breast cancer (*BRCA*) genes. Another interaction between *BRCA1a* and *BRCA1b* had been reported in an earlier study by Chai et al. [92]. In the study it is described how *BRCA1a/b* may target *ELK1* and thus suppresses ELK1-mediated gene transcription, affecting genes like *FOS*, and therefore, limiting BC cell growth [92]. Another study focused on a potential tumor-suppressing role of 15-deoxy-Δ(12,14)-prostaglandin J2 (15 d-PGJ2). 15 d-PGJ2 was found to promote the expression 15-Hydroxyprostaglandin dehydrogenase (15-PGDH) enzyme (which converts prostaglandin E2 (PGE2) into an inactive form via activation of ERK1/2 and ELK1 [93]. Since PGE2 can promote BC tumorigenesis, the study concluded that 15 d-PGJ2 may activate ELK1, but instead of attributing ELK1’d expression with tumorigenic activity, its activation may result in tumor-suppressive effects [93]. A 2007 study reports that tamoxifen activates the expression of p21^Waf1/Cip1^ via a transient activation of EGR1 [94]. The upregulation of Early growth response 1 (EGR1) was found to be a result of ELK1’s activation via the JNK and p38 MAPKs, which were herein found to promote the expression of factors promoting cell cycle arrest instead of proliferation [94]. The activation of p21 in an ELK1 dependent manner has also been described elsewhere, implicate a transcription axis of increased pharmaceutical significance [95].

Several studies have investigated how cytostatic phenomena are correlated to ELK1, both in the context of transcriptional regulation of the TF’s expression, as well as in the context of its activation by upstream kinases. A 2016 study by Kole et al., it was demonstrated how Pioglitazone (Pio), which belongs to a class of Peroxisome proliferator-activated receptor gamma (PPAR-γ) ligands, can suppresses the proliferation of MCF-7 cells [96]. The authors reported that Pio induces the expression of p21c^ip1/waf1^/p27^kip1^ and downregulates CDK4, via the sustained activation of MAPKs which activates ELK1- mediated gene transcription [96]. Another study focused on fucoxanthin, a marine carotenoid and revealed that it modulates the expression of several oncogenic genes [97]. Fucoxanthin was found to downregulate *ELK1* mRNA in both MDA-MB-231 and MDA-MB-468, while in MDA-MB-231 the protein levels of ELK1 also decreased [97]. Mechanistically, the main mechanism of action was theorized to be a modulation of PI3K/Akt mediated signaling, which was shown to affect key cellular functions [97]. Another study investigated how the sensitivity of BC cells to cisplatin can be improved and revealed that Letrozole, an aromatase inhibitor can improve response to cisplatin by targeting FEN1 [98]. BC cells that overexpressed aromatase, were found to downregulate FEN1 following treatment with Letrozole, in a mechanism that involved the MAPK-ELK1 pathway [98]. Letrozole was able to inhibit the activation of ELK1 induced by testosterone, indicating how the suppression of FEN1 expression can be targeted by MAPK-ELK1 inhibition [98].

A 2005 study described how Glucocorticoid receptor (GR) activation can halt apoptosis induced by chemotherapeutics like paclitaxel [99]. Wu et al. showed that its activation promotes the expression of MAPK phosphatase-1 (MKP-1; also known as Dual specificity phosphatase 1/DUSP1) and downregulates the phosphorylation of ELK1 [99]. Even though ELK1 dephosphorylation can have detrimental effects on cell viability, ELK1’s activation by sustained MAPK activation (both ERKs and JNKs) has proapoptotic effects [96,100,101]; therefore, dexamethasone treatment which leads to GR activation, exhibits cytoprotective roles [99]. The study also described that human tissue plasminogen activator (tPA) is a target of ELK1-mediated gene transcription [99], further supporting the notion of ELK1 targeting as part of anticancer therapies, given the pro-tumor roles attributed to Plasminogen activator, tissue type (tPA or PLAT). Regarding ELK1’s proapoptotic roles, a 2014 study investigated the anticancer effects of Withaferin A (WFA) on BC and reports that WFA promotes the phosphorylation of RSK1 [102] (Table 2). Following RSK1 activation, ELK1 was also reported to be activated, and together with ERK-RSK and DNA damage-inducible transcript 3 protein (DDIT3; also known as C/EBP homologous protein, CHOP) were found to upregulate the expression of Death receptor 5 (DR5) [102]. This is another example of pro-apoptotic activity and it has been reported elsewhere [103], indicating pathways that could be pharmaceutically targeted to sensitize cancer cells to apoptosis [102].

**Table 2:** Pharmaceuticals targeting ELK1 in lung, breast, and colorectal cancer.

Cancer	Substance	Sample/Model	Effect(s) on ELK1	Mechanism	Outcome	Ref.
NSCLC	Hexagonal selenium nanoparticles modified by siRNA (HSNM-siRNA)	Human NSCLC cell lines (A549, H1299, H520, and H1975)	Decrease in ELK1 expression	Inhibition of EGFR signaling	Cell cycle arrest, reduced viability, and apoptosis induction	[46]
NSCLC	Afatinib	Patient-derived tissues (47 NSCLC patients) and human NSCLC cell lines (H358, H441, A549 H460)	Decrease in ELK1 phosphorylation	Downregulation of CIP2A, promotion of PP2A activity, and decrease in AKT phosphorylation	Apoptosis induction	[49]
BC	Withaferin A (WFA)	Human BC cell lines (MCF-7, MDA-MB-231, T-47D, and MDA-MB-468) and mice xenografts	Increase in ELK1 phosphorylation	Upregulation of Death receptor 5 (DR5) expression	Apoptosis induction	[102]
BC	Grifolin	Human BC cell lines (MCF-7)	Decrease in ELK1 phosphorylation	Direct inhibition of ERK1/2 activity	Decrease in cell adhesion, migration and invasion	[104]
CRC	Curcumin	Human colon cancer cell lines (Moser cells, Caco-2 and HT-29)	Decrease in ELK1 expression	Reduction in the transcriptional activity of EGR1	Reduced viability	[105]
CRC	6-(Methyl-sulfinyl)-hexyl isothiocyanate (6-MSITC)	Human CRC cell line (HT-116)	Increase in ELK1 phosphorylation	Overexpression of DNA damage-inducible transcript 3 protein (DDIT3) and Death receptor 5 (DR5)	Apoptosis induction	[103]
CRC	Monensin	Human CRC cell lines (RKO and HCT-116)	Decrease in ELK1 phosphorylation	Inhibition of IGFR signaling	Decrease in cell cycle progression, proliferation, migration, and invasion	[106]
CRC	Gossypol	Human colon cancer cell line (COLO 205-ATCC CCL-222)	Decrease in ELK1 expression	Suppression of *CLAUDIN1*, *ELK1*, *FAS*, *GAPDH*, *IL2*, *IL8*, and *ZFAND5* and upregulation of *GLUT3*	Reduced the viability	[107]
CRC	CYC202 (R-roscovitine)	Human CRC cell lines (HT-29, NIH3T3, and KM-12)	Increase in ELK1 phosphorylation	Inhibition of transcription, possibly via the inhibition of both CDK7 and CDK9 complexes	Cell cycle arrest	[108]

Abbreviations: NSCLC = Non-small cell lung cancer; BC = Breast cancer; CRC = Colorectal cancer.

Finally, ELK1 was studied in the context of tamoxifen resistance, as part of positive feedback loop that leads to upregulated EGR1 signaling [109]. According to Marks et al., upon treatment of MCF-7 cells with Recombinant glial cell-derived neurotrophic factor (rGDNF), and induction of Rearranged-during-transfection (RET) tyrosine kinase signaling was documented which led to the activation of ELK1 [109]. ELK1 leads to EGR1 expression, which in turns upregulates the expression of several cell-cycle proteins, mainly Cyclin D1, and leads to increased cell proliferation [109].

### 2.3. Colorectal Cancer

ELK1 has been reported several times in the context of Colorectal carcinoma (CRC). ELK1 has been found to be a cornerstone of CRC pro-metastatic, and in general pro-EMT, signaling. A 2017 study by Zhao et al. reported that CRC tumors overexpress the chemokine C-X-C motif chemokine ligand 5 (CXCL5), which is also a poor prognosis factor and an EMT driver [110]. CXCL5 was reported to act by activating Snail family transcriptional repressor 1 (Snail or SNAI1) via the ERK/ELK1/SNAI1 pathway upon stimulation of the C-X-C motif chemokine receptor 2 (CXCR2), thus crediting ELK1 with an important role in EMT tuning and progression [110]. Regarding chemokine signaling, C-C motif chemokine ligand 19 (CCL19) is another example of ELK1’s participation in chemokine-initiated signaling [111]. CCL19 was reported to be downregulated in CRC, while its overexpression using in vitro experiments results in proliferation, migration, and angiogenesis [111]. CCL19 was proposed to act by promoting miR-206, following stimulation through the C-C motif chemokine receptor 7 (CCR7) [111]. Acceding to Xu et al., miR-206 inhibits ELK1 (as part of a broader axis involving MET/ERK/ELK1/HIF-1α/VEGF-A) [111]. ELK1’s expression has also been shown to be affected by CRC cell exposure to Lipopolysaccharide (LPS), from the outer part of the bacterial cell walls [112]. A 2023 study by Cao reports that LPS does not affect the viability of COLO225 cells; however, the expression of *ELK1* mRNA is upregulated [112]. Ma et al. in 2021 reported that in CRC, c-KIT facilitates ELK1 activation via the MAPK pathway [113]. Additionally, ELK1 was found to be positively correlated to Carcinoembryonic antigen-related cell adhesion molecule 5 (CEACAM5 or simply CEA) expression and all three proteins (KIT, ELK1, CEA) were reported to be upregulated in CRC patients, highlighting the importance of the KIT/MAPK/ELK1/CEA axis as prognostic/diagnostic marker and as a potential pharmaceutical target [113]. The expression of Fucosyltransferase 1 (FUT1) has also been found to be ELK1-dependant [114]. FUT1 has been reported to be a marker of tumor stemness [115], thus crediting ELK1 with a significant regulatory aspect in this context. A 2005 study investigated how 17beta-Estradiol (E2) can induce the proliferation of colonic carcinoma cells [116]. The authors reported that E2 caused significant increases in CREB and ELK1 phosphorylation, which was found to be MAPK mediated [116]. These actions of E2 were described as non-genomic and led to *FOS* gene transcription which was responsible for the increased proliferation [116]. However, an estrogen receptor (ER) antagonist, inhibited the activation of ELK1 and CREB, indicating that the process was ER-dependent [116]. A recent study (2025) identified ELK1 and MYC as a targets of RPL36A-mediated signaling [117]. Ribosomal protein L36A (RPL36A) is overexpressed in CRC and this study by Shi et al. reported that upon RPL36A silencing, tumor progression was halted a decrease in ERK1/2 and ELK1 activation was documented [117]. Another protein transducing signal via ELK1 in CRC has been reported to be A-kinase anchor protein 95 (AKAP95) [118]. Kong et al. in 2023 reported that AKAP95 is correlated to the activation of the BRAF-MEK-MAPK pathway, acting via phosphorylation of ELK1 and significantly affects immune cell infiltration and colon cancer patient survival [118]. ELK1’s role in CRC tumorigenesis and metastasis was also reported in a 2025 study describing how it can regulate the expression of ADAMTSs, a class of inflammation-related matrix metalloproteinases [19]. The expression of ADAMTS-8, which has been correlated to poot prognosis, was found to be controlled by IL6 stimulation via the activation of STAT3, c-JUN, and ELK1 [19]. ELK1 has also been found to be correlated to COX-2 expression.

*KRAS* mutations are an important parameter affecting ELK1 activity in CRC. A recent study investigated how different *RAS* mutations can regulate downstream MAPK signaling in CRC in Filipino patients [119]. Following comparison of KRAS^G12D^, KRAS^G12S^, KRAS^A59T^, KRAS^Y137C^, NRAS^G12D^, and NRAS^A11V^, only KRAS^G12S^ and KRAS^A59T^ were reported to be MAPK-signaling deregulators affect the activation of ELK1 [119]. However, it is noteworthy that only Filipino patients were studied; therefore, this could reflect this particular demographic. Studies of KRAS activity and correlation with ELK1 activity have been conducted again in the past. Modest et al. in 2013 reported that KRAS contributes to sunitinib resistance and that *KRAS* alleles determine the grade of resistance [120]. Using ERK activation as an index, KRAS ^G12V^ was found to be the most susceptible to sunitinib, while KRAS ^G12A^ was correlated to the least ERK phosphorylation activity [120]. Cells with wild type KRAS, exhibited greater decreases in ELK1 phosphorylation compared to all mutation, indicating that a mutant KRAS protein is a significant contributor to ELK1’s activity, thus underscoring how KRAS mutant tumors may acquire resistance to TKIs in a more rapid rate [120]. A 2016 study by Hollander et al., compiled a method that integrated physical interactions, gene expression patterns, and alternative splicing data aiming to study cancerous splicing aberrations and identify potential pathways with a driver role.

There is also data reporting the pivotal role of ELK1 from a systems biology approach. ELK1 was identified as a key component of the network, acting primarily by promoting MYC’s transcription, as well as by inducing the alternative splicing factor Polypyrimidine tract binding protein 1 (PTBP1), together with c-MYC [121]. Its pivotal role in tumorigenesis is further reinforced in KRAS-mutant tumors, in which, continuous MAPK activation leads to ELK1-mediated phenomena, including gene transcription and triggering of alternative splicing [121]. Another study focused on identifying TFs with crucial roles in CRC pathogenesis [122]. ELK1 was reported to be the most significant among a set of TFs (SNAI2, RUNX1, Interferon regulatory factor 1 (IRF1), HIF-1A, Activating transcription factor 2 (ATF2 or CREB2), ABL proto-oncogene 1 (ABL1), ELK1, and GATA binding protein 1 (GATA1)), since its interaction with JNK1 was suggested as a significant marker in CRC tumorigenesis and progression [122].

ELK1 has been credited with pivotal roles in the regulation of CRC progression via its interaction with the expression of noncoding RNAs. ELK1-regulated noncoding RNAs in CRC have been implicated in multiple cancer hallmarks, including EMT, TME remodeling, and metabolic adaptation. Regarding EMT, one such example is the regulation of the pro-tumorigenic micro-RNA miR-31-5p, an RNA molecule implicated in metastasis, regulation of autophagy, and apoptosis [123]. Yan and Lei in 2023 reported that ELK1 mediates the expression of miR-31-5p, which in turns act by targeting the Cell death inducing P53 target 1 (*CDIP*) gene expression [123]. ELK1 has also been reported to be the target of micro-RNAs that act as tumor suppressors. Fan et al. reported that low miR-873 expression is a poor prognosis factor for CRC, since the proliferation and migration of miR-873-deficient cells is augmented [124]. miR-873 was shown to directly bind to the 3′UTR of ELK1 and Striatin-4/Calmodulin binding protein 4 (*STRN4*) mRNAs, inhibiting their translation and thus silencing ELK1’s tumorigenic activity [124]. A 2022 study revealed that Circ_0022340 can sponge the micro-RNA miR-382-5p and thus promote ELK1’s expression, since miR-382-5p targets the ELK1 mRNA [125]. This mechanism underscores how ELK1 undergoes post-transcriptional regulation in CRC cells and highlighted the role of Circ_0022340 or miR-382-5p as potential targets to modulate its activity [125]. A significant regulatory role regarding EMT-related roles is also exhibited by the lncRNA LBX2-AS1 [37]. It is reported to be an independent poor prognosis factor in CRC, and a potential diagnostic marker, the transcription of which is regulated by ELK1 [37]. LBX2-AS1 was found to act by blocking the degradation of S100 calcium binding protein A11 (S100A11 or MLN70) or by sponging the tumor-suppressing micro-RNA miR-491-5p, both mechanisms promoting cell proliferation, migration, and invasion [37]. Concerning Tumor microenvironment (TME) remodeling, a study reported that CRC metastatic cells release the pro-metastatic miR-181a-5p, which targets the Suppressor of cytokine signaling 3 (*SOCS3*) gene and activates STAT3-mediated-signaling [126]. The expression of miR-181a-5p was shown to be regulated by ELK1, which is activated by the C-C motif chemokine ligand 20 (CCL20), via the C-C motif chemokine receptor 6 (CCR6)/MAPK pathway [126]. This mechanism seems to form a positive loop since highly metastatic cells are able to both stimulate chemokine signaling and get activated by it. The study also reported that CRC cells pack miR-181a-5p into extracellular vesicles that target cells in the tumor microenvironment and thus tune their signaling pathways facilitate TME remodeling [126]. Regarding TME, a recent study (2025) by Chen et al., analyzed 751,531 single-cell transcriptomes, spatial transcriptomics, and snMultiomes from 142 multistage samples and revealed that the expression of Galactin-9/Lectin galactoside-binding soluble 9 (LGALS9), which is regulated by ELK1, promotes the transformation of fibroblasts near CRC tumors, into cancer-associated fibroblasts, further fueling TME remodeling and tumor progression [127]. Finally, ELK1-mediated RNA regulation of metabolism has also been reported. A 2021 study investigated how the long noncoding RNA MIR17HG, which is a poor prognostic factor, contributes to metabolic adaptation of CRC cells [128]. The authors report that MIR17HG, the transcription of which is controlled by ELK1, promotes glycolysis via the sponging of the miR-138-5p, a micro-RNA that normally downregulates Hexokinase A (HK1) translation [128]. The involvement of MIR17HG results in intracellular lactate accumulation which has been found to activate ELK1 by triggering p38 MAPK signaling [128].

Downregulation or targeting of the ELK1-mediated transcription has been shown to effectively inhibit CRC cell proliferation, migration, and invasion activity. Recently, the C4orf19 protein was found to downregulate ELK1’s activity in an indirect axis involving: the E3 ligase Kelch-like ECH-associated protein 1 (KEAP1); the ubiquitination mediated by Tripartite motif-containing protein 25 (TRIM25; also known as E3 ubiquitin/ISG15 ligase); and the deubiquitinating enzyme USP17 [129]. Chromosome 4 open reading frame 19 (C4orf19) was found to inhibit the degradation of KEAP1, the accumulation of which leads to the degradation of Ubiquitin specific peptidase 17 like protein 2 (USP17) [129]. USP17’s degradation induces ELK1’ degradation thus reducing its ability to facilitate the transcription of pro-proliferation proteins like Cyclin-dependent kinase 6 (CDK6) [129]. A 2005 study investigated whether curcumin could suppress CRC and reported that upon treatment with it, the viability of CRC cells reduces [130]. A study by the same group demonstrated that curcumin acts by reducing the transcriptional activity of EGR1 [105]. This reduction in EGR1 activity inhibits the expression of both EGFR and ELK1, being the uppermost and nethermost component of the MAPK pathway, and thus, completely blocks pro-survival signal transduction [105]. Another study explored the anticancer potential 6-(Methylsulfinyl)hexyl isothiocyanate (6-MSITC) against CRC cells [103]. 6-MSITC was found to induce apoptosis by stimulating the overexpression of DNA damage-inducible transcript 3 protein (DDIT3; also known as C/EBP homologous protein, CHOP) and Death receptor 5 (DR5) [103]. Mechanistically, the authors suggest that 6-MSITC increased pro-apoptotic gene transcription by ERK1/2 and ELK1 activation [103]. Although ERK1/2 signaling is mostly involved in pro-survival mechanisms (in both CRC and other cell types), the authors also report that inhibition of MEK1/2 using the U0126 inhibitor, reversed the effects of 6-MSITC [103]. A 2023 study investigated the anticancer activities of monensin against CRC and reported that upon treatment with the antibiotic, cell cycle progression, proliferation, migration, and invasion decrease [106]. From a mechanistical point of view the authors explored how IGF signaling is affected and showed that ELK1, c-MYC, AP-1 were activity was diminished, while the expression of IGF1R was also suppressed [106]. In a 2021 study by Cao et al. showed that gossypol, a plant phenol, can reduce the viability of human colon cancer cells by downregulating ELK1 [107]. Another approach was published in a 2013 study, describing how the targeting of Dynein light chain roadblock-type 1 (DYNLRB1; also known as km23-1)in human CRC cells can suppress migration and invasion [131]. DYNLRB1 is an actin modulator, but also able to affect the RAS-RAF-MEK-MAPK pathway in TGFβ-sensitive epithelial cells [131]. This study by Jin et al. showed that DYNLRB1 silencing activates ERK1/2-mediated signaling, inhibits the phosphorylation of ELK1 and TGF-β1 and Ezrin (EZR; also known as Cytovillin, Villin 2) synthesis [131]. These effects have profound antimetastatic potential, highlighting how indirect ELK1 targeting can be part of a therapeutic approach against CRC [131]. Another strategy is described in Whittaker et al. (2004), in which the cyclin-dependent kinase inhibitor CYC202 (R-roscovitine) was investigated for its potential anti-CRC actions [108]. CYC202 was documented to decrease the expression and phosphorylation levels of the retinoblastoma protein, and an increase in ERK1/2 phosphorylation which led to ELK1 and FOS activation [108]. Regardless of the activation of the MAPK that serve as pro-survival signal transducers, the expression of Cyclins D1 (CCND1), A (CCNA), and B1 (CCNB1) reduced significantly [108]. The role of ELK1 was not studied further; however, its activation was not adequate to overcome the cell arrest signaling or promote the transcription of the suppressed cyclins.

The phosphorylation status of ELK1 has been a matter of research as well. Phosphorylation at different sites was reported to lead to differential results regarding its activity [132]. A 2015 study reports that phosphorylation at Thr^417^ can be used as marker of differentiation in multiple markers [132]. The study reports that regarding colonic adenocarcinoma, a higher percentage of ELK1 p-Thr^417^ positive cells indicates a well-differentiated adenocarcinoma, while both normal colon or poorly differentiated adenocarcinoma exhibit lower percentages of such cells [132]. This was further confirmed in a 2022 study, in which poorly differentiated CRC tumors were reported to downregulate ELK1 levels in comparison to moderately differentiated ones [133]. Finally, in another 2011 study, CRC tumors with high Prostaglandin-endoperoxide synthase 2 (PTGS2; also known as Cyclooxygenase-2, COX2) expression were also documented to have decreased ELK1 expression [134]. The study mentions that PTGS2 is a marker of CRC progression [134]; therefore, decreased ELK1 expression could be a marker for an advanced stage disease, as has also been demonstrated by other researchers [133].

ELK1 has also been reported as key TF promoting chemoresistance. A recent study investigated 5-Fluorouracil (5-FU) resistance and focused on the metabolic adaptations of 5-FU-resistant CRC cells [135]. Serine deprivation was found to promote the expression of Serine synthesis pathway (SSP) genes, the transcription of which was shown to be indirectly modulated by ELK1, through activation of the MAPK pathway [135]. The authors report that ELK1 is not the TF activating SSPs; however, Forkhead box C1 (*FOXC1*), which is a target of ELK1-mediated transcription is the responsible TF, and suggest that targeting the ELK1-FOXC1 axis could be another potential approach against 5-FU-resistant CRC tumors [135].

### 2.4. Prostate Cancer

In prostate cancer (PCa), ELK1 has been credited with pivotal roles regarding tumorigenesis, tumor progression, and metastasis. ELK1 has been found to participate in several transcriptional axes, engaging in extensive crosstalk with AR-related signaling, and to be a significant contributor to drug resistance. A 2020 study identified ELK1 as an independent prognostic marker of disease recurrence in PCa [136]. More importantly, compared to the other ELK TF family members (ELK3 and ELK4), only ELK1 was associated with disease-free survival (DFS), thus highlighting its significance in the underlying mechanisms of PCa progression [136]. A computational study on DNase sequencing data (GSE33216) and RNA sequencing data (GSE22260) from the Gene Expression Omnibus database, found that ELK1 and CCCTC-binding factor (CTCF) are significantly downregulated in LNCaP cells and DHT-treated LNCaP cells [137].

In 2013, Patki et al. reported that in AR-dependent PCa cells, ELK1’s expression is obligatory for cell growth, while AR-independent cells do not rely on ELK1 [138]. A 2014 study investigate which TFs can assist adaptation to androgen deprivation [139]. ELK1, among others (EVI1, NFY, GATA2, MYBL1, MYBL2, and NFκβ family members (NFκβ1, NFκβ2, REL, RELA and RELB) and highlighted how this upregulation assists cell survival and orchestrates the emergence of castration-resistant PCa [139]. Another study, by Rosati et al. targeted the activation of ELK1 by AR and investigated how this inhibition can affect androgen-dependent cell lines [140]. Upon treatment with KCI807, that disrupts ELK1-AR interactions but not the ERK1/2-mediated phosphorylation of ELK1, the growth of the cells declined, both in castration/enzalutamide-resistant cell lines and patient-derived tumor xenografts (Table 3) [140]. The exact mechanisms underlying KCI807’s activity and the relevant biding sites were further investigated in a recent study by Soave et al. in HeLa-HLR cells [11].

Several studies were also conducted to define the ELK1-AR interaction from a structural-biology perspective. A 2016 study by Rosati et al., identified both ERK- and AR-activated motifs, as well as exclusively ERK-dependent motifs [141]. The D-box and the DEF (docking site for ERK, FXFP) motif, were identified as two ERK-docking sites in ELK1 which were also found to be the essential motifs for its interaction with AR(A/B) or WTAR [141]. On the contrary, the transactivation domain in ELK1 was identified to be solely ERK-dependent. The study reported that mutant ELK1 in which the D-box and DEF motifs were disrupted did not interact with AR, while mutant-ELK1 with deletion of the D-box region, had a dominant-negative effect on AR-dependent growth of PCa cells [141]. Deletion of the D-box in ELK1 made the cells unresponsive to MAPK pathway inhibitors, further supporting the importance of AR-ELK1 interaction in AR-dependent cells [141]. A 2022 study by Soave et al. identified two peptide segments in the AR protein, that mediate its association with two ERK1/2-docking sites on ELK1 [142]. The authors validated their approach by deleting either site on the AR, thus effectively inhibiting its interaction with ELK1 [142]. The lack of interaction between AR and ELK1 led to decreased PCa cell growth and significantly impaired key cellular functions [142]. Another study investigated how Flap endonuclease 1 (FEN1) affects resistance against docetaxel (DTX) in PCa cell lines and reports that ELK1 regulates its expression [143]. AR activation was found to upregulate p-ELK1 which in turn promoted the transcription of FEN1, significantly suppressing DTX-induced apoptosis and cell cycle arrest [143]. Recently, in PCa, ELK1 was reported to cooperate with MED19 to regulate the transcription of AR and AR co-regulators [144]. More specifically, at androgen deprivation therapy (ADT) conditions, ELK1 and MED19 were shown to regulate the same set of genes [144]. The ELK1-MED19 cooperation was shown to regulate the occupancy of AR and control the expression of monoamine oxidase A (MAOA), an enzyme correlated to androgen-independent growth [144].

The activation of ELK1 by growth factor signaling in PCa cells has also been a matter of research for several years. Azami et al. reported that EGFR signaling via the RAS-RAF-MEK-MAPK pathway can activate ELK1, which in turn regulates the expression of Transmembrane prostate androgen induced-RNA/Prostate transmembrane protein, androgen induced 1 (TMEPAI/PMEPA1) [145]. Even though TMEPAI had been reported in the past to be a downstream target of TGF-β signaling, via Smad3 activation, EGFR activation was found to be another regulatory mechanism, working synergistically to promote the MAPK-ELK1-dependent transcription of TMEPAI which has known oncogenic activity [145]. Additionally, in the context of EGF-initiated growth, ELK1 was found to be activated via the RAS-RAF-MEK-MAPK pathway and promote the expression of EGR1 [146]. Finally, data also exists on the inhibition of growth-factor-initiated signaling and the effects on ELK1’s activation. The expression of the tumor suppressor gene NKX3.1 was found to downregulate IGF-I-mediated cell growth, and the activation of ERK1/2, ELK1, and suppressed the expression of IGF-1R, Cyclin D1, and c-FOS [147]. Another study even reported that the androgens 5α-dihydrotestosterone and R1881 can suppress the EGF-initiated/ELK1-mediated transcription of the FOS gene, by activation of PKCδ and the MAPK p38 [148]. A 2012 study by Sen et al., investigated how Paxillin participates in ELK1 activation [149]. The study reported that Paxillin regulates both androgen- and EGF-dependent signaling [149], acting as an integration point. Upon stimulation with androgen and EGF, MAPK-dependent phosphorylation of Paxillin was observed as well as translocation to the nucleus [149]. It was found associating with both AR and complexes of ERK1/2-ELK1, thus inducing the expression of c-Fos and cyclin D1, both of which promote proliferation [149]. Regarding the regulation of ELK1’s expression, was found to be dependent on ETS1 in PC-3 cells [150].

Several mechanisms of pro-tumorigenic activity have been correlated to ELK1 over the years. A recent study by Cui et al., investigated how castration resistant prostate cancer cells, can be stimulated to express 3β-Hydroxysteroid dehydrogenase-1 (3βHSD1), the enzyme catalyzing the synthesis of extragonadal androgens [151]. The study revealed that cancer-associated fibroblasts (CAFs), via the excretion of glucosamine, activate ELK1, which in turn activates the transcription of the 3βHSD gene [151]. A 2022 study reported that ELK1 regulates the expression of YTH m6A RNA-binding protein 1 (YTHDF1) [152], which is a protein participating in post-transcriptional modifications of RNA molecules that induce their translation and stability [153]. The study reported that by mediating YTHDF1’s overexpression, ELK1 is implicated in the expression of polo-like kinase 1 (PLK1), which promotes cell cycle progression and contributed to tumor growth and chemoresistance [152]. Additionally, ELK1 has been found to control the expression of AIRE in androgen-independent cells, and through AIRE, the cytokine signaling [154]. According to Kalra et al., AIRE regulates the expression of IL-6 and aspects of the tumor microenvironment (TME), thus fueling survival pathways implicated in tumor progression and chemoresistance [154]. Another study explored the pro-metastatic role of ELK1 via its participation in PRK1-SPAG9-p38-mediated signaling as the nethermost effector [155]. ELK1 silencing was reported to inhibit the pro-migratory signaling by PRK1while PRK1 overexpression (and an active ELK1) lead to increased migration and transcriptional activity [155]. An older study, conducted in 2002 showed that Bombesin can induce the activation of ELK1 by stimulating the gastrin-releasing peptide receptor (GRP-R), which lead to an expression of the *JUN* gene [156]. In a 2004 study, ELK1 was reported to be activated by the prosaptide TX14A, a neurotrophic factor, which activated RAS-RAF-MEK-MAPK signaling in androgen-independent PCa cell lines [157]. A recent study focused on the regulation of the protein Stomatin (STOM) in PCa, the expression of which was reported to be dependent on ELK1’s activation [158]. The authors also reported that ephrin-A5 (EFNA5) stimulates the receptors EPH receptor A3 (EPHA3) and EPH receptor A7 (EPHA7) to induce ERK1/2 phosphorylation and the subsequent activation of ELK1 and ELK4 [158]. In other studies, ELK1 was reported to be a target of the TNF/IL-1 signaling cascade, and mediate pro-proliferation transcriptional activity, thus affecting tumorigenesis and PCa progression [159,160].

ELK1 has also been reported in the context of noncoding RNA regulation. A 2013 study by Wang et al. investigate how the expression of the micro-RNA let-7a affects PCa cell growth [161]. miRNA let-7a was found to negatively regulate the expression of IGF1R and leads to decreased ELK1 activation and c-FOS expression [161]. A computational study on microarray data from the GSE28029 dataset in the GEO database investigated potential micro-RNA targets from the TargetScanHuman, miRDB and DIANA microT databases. ELK1 was one of the six most common genes (with the others being FOXC1, CDKN1A, BIRC2, CTNND1, and LRP8) all of which were found to be upregulated in PCa, thus emphasizing their pivotal role in tumorigenesis and their regulation by MicroRNAs [162].

A 2003 study reported also reported that treatment of PC-3 and LNCaP cells with quercetin inhibited cell growth and the phosphorylation of ELK1 among other proteins of the MAPK (RAF1, MEK1/2, and ERK1/2) and AKT pathways [163]. A 2016 study by Kawahara et al. investigated how silodosin affects ELK1-mediated cell growth of both AR-dependent and AR-independent PCa cell lines [164]. Kawahara et al. reports that ELK1 is overexpressed in carcinoma tissues (compared to both benign hyperplasia of the gland and high-grade prostatic intraepithelial neoplasia) and the protein acts as a marker of poor prognosis [164]. ELK1 silencing was reported to decrease cell migration and invasion but not viability in AR-independent cell lines [164]. Upon treatment with silodosin, the authors reported that the expression and the phosphorylation of ELK1 are reduced, which leads to a decline in all cells’ migratory abilities (both AR-dependent and AR-independent cell lines) [164]. Additionally, in AR-dependent cells, ELK1 downregulation due to silodosin was reported to decrease cell viability [164]. Silodosin’s effects on prostate tissue (smooth muscle) had been reported in a 2012 study, in which it was reported that silodosin can inhibit ELK1’s activation (induced by noradrenaline or phenylephrine) and thus reduce its activity [165]. In another study, the effects of silibidin on PCa cells were studied [166]. Silibidin was found to decrease the protein levels of TGFα and the phosphorylation levels of EGFR [166]. Decreased activation of ERK1/2 and ELK1 was also reported, thus suggesting that silibidin may decrease the activity of the autocrine TGFα/EGFR signaling pathway, by targeting the ELK1-mediated transcription of TGFα [166]. This mechanism has also been found to be the target of flavonoid procyanides, contained in grape seed extract (GSE) [167]. GSE was found to reduce ERK1/2 and ELK1 phosphorylation levels; however, the activation of JNK1/2 was found to be elevated, leading to an increase in c-Jun’s activity [167]. This mechanism was accredited with pro-apoptotic roles and was theorized to further enhance the effects of ELK1’s decreased phosphorylation caused by EGFR signaling inhibition [167]. A 2008 study identified ELK1 as one of the targets of Compound A (CpdA), a stable analog of an aziridine precursor from the African shrub *Salsola tuberculatiformis Botschantzev* [168]. CpdA was reported to target both glucocorticoid receptor (GR)- and AR-mediated signaling thus inhibiting the activation of NF-κΒ, AP-1, ETS1, ELK1, SRF, CRE/ATF, and NFATc [168]. Asiatic acid was recently reported as an anti-metastatic agent in metastatic PCa cell lines and Lai et al. reported that its actions were mediated by inhibiting the activity of SNAI1 [169]. Treatment with Asiatic acid was found to impair the ELK1/MZF1 interaction thus reducing the transcription of SNAIL [169]. Finally, ELK1 has also been reported as one of the targets of *Phyllanthus* (*P. amarus*, *P. niruri*, *P. urinaria*, and *P. watsonii*) plant extracts [170]. The plant extracts were found to significantly impair RAS-RAF-MEK-MAPK-ELK1 signaling, as well as other pathways implicated in cell adhesion, apoptosis, metastasis, angiogenesis, and metabolism [170].

**Table 3:** Pharmaceuticals targeting ELK1 in prostate cancer.

Cancer	Substance	Model/Sample	Effect(s) on ELK1	Mechanism	Outcome	Ref.
PCa	KCI807	Human PCa cell lines (LNCaP, VCaP, 22Rv1) and mice xenografts	ELK1-AR interaction disruption	Binding to AR, blocks the ELK1 binding and the ELK1-mediated recruitment of AR to chromatin	Growth inhibition	[140]
PCa	Quercetin	Human PCa cell lines (PC-3 and LNCaP)	Decrease in ELK1 phosphorylation	Modulation of MAPK- and AKT-associated signaling	Growth inhibition	[163]
PCa	Silodosin	Patient-derived tissues (150 PCa patients) and human PCa cell lines (PC-3, DU-145, LNCaP, and C4-2)	Decrease in ELK1 expression and phosphorylation	Selectively blocking of α1A-adrenergic receptors	Reduced migration	[164]
PCa	Silibidin	Human PCa cell lines (DU-145 and LNCaP)	Decrease in ELK1 phosphorylation	Inhibition of TGFα/EGFR signaling	Decreases in secreted and cellular TGFα	[166]
PCa	Procyanides (as parts of grape seed extract)	Human PCa cell line (DU-145)	Decrease in ELK1 phosphorylation	Inhibition of EGFR signaling and activation of JNK/c-Jun.	Apoptosis induction	[167]
PCa	Compound A (CpdA),	Human PCa cell lines (PC-3, DU-145 and LNCaP)	Decrease in ELK1 phosphorylation	Targeting of GR- and AR-mediated signaling via the inhibition of NF-κΒ, AP-1, ETS1, ELK1, SRF, CRE/ATF, and NFATc	Growth inhibition	[168]
PCa	Asiatic acid	Human PCa cell lines (PC-3, DU-145 and 22Rv1)	ELK1-MZF1 interaction disruption	Reduced transcription of SNAIL	Reduced migration	[169]
PCa	*Phyllanthus* plant extracts	Human PCa cell line (PC-3)	Decrease in ELK1 phosphorylation	Inhibition of RAS-RAF-MEK-MAPK-ELK1 signaling	Impairment of cell adhesion, apoptosis, metastasis, angiogenesis, and metabolism	[170]
PCa	Sulforaphane (SFN), phenethyl isothiocyanate (PEITC) and allyl isothiocyanate (AITC)	Human PCa cell line (PC-3)	Increase in ELK1 phosphorylation	ERK- and JNK-dependent activation of AP-1	Reduced viability	[171]
PCa	Parthenolide (PTL)	Human PCa cell lines (PC-3, DU-145, VCaP and LAPC4), primary prostate TICs, and mice models	Decrease in ELK1 expression	Decrease in the levels of ELK1, FGFR2, PKCs, MEKs, MAPKs, CaMs	Reduced viability	[172]

Abbreviations: PCa = Prostate cancer.

ELK1 has also been reported to participate in cancer chemoprevention. A 2006 study reported that the isothiocyanates (ITCs) sulforaphane (SFN), phenethyl isothiocyanate (PEITC) and allyl isothiocyanate (AITC) exhibit anticancer activities by activating ERK1/2, JNK1/2, c-JUN, and ELK1, as well as AP-1 [171]. The study concluded that ITCs reduced the expression of BCL2 and lead to decreases in cell viability, which are both ERK- and JNK-dependent, through the activation of AP-1 [171]. A 2009 study by Kawasaki et al. showed that ELK1 and a set of other genes (FGFR2, PKC, MEK/MAPK, CaM) are direct targets of the sesquiterpene lactone parthenolide (PTL) [172]. PTL was found to be particularly cytotoxic to prostatic cancer stem cells (or tumor-initiating cells, TICs), thus underscoring ELK1’s role in early PCa tumorigenesis and how its targeting may be a potential approach [172].

Finally, ELK1 has also been reported in drug resistance and sensitivity. A recent study reported ELK1 being upregulated in bortezomib-resistant PCa cells, both in terms of expression and phosphorylation [20]. ELK1’s activation was a result of increased MAPK activation, which has been reported repeatedly in the context of proteasome-inhibitor-resistance [20,173]. A 2016 study reported that the downregulation of ELK1’s phosphorylation, as a result of silodosin’s activity, can increase the chemosensitivity to gemcitabine, in both AR-dependent and -independent PCa cells [164]. On the other hand, silodosin, regardless of the interference with ELK1-mediated transcription, was not shown to affect the sensitivity to cisplatin or docetaxel [164]. However, given the fact that ELK1 has been found implicated in both paclitaxel and cisplatin/oxaliplatin resistance, these mechanisms may be tissue/cancer-specific [98,174,175,176,177,178].

### 2.5. Gastric Cancer

**Figure 1:**
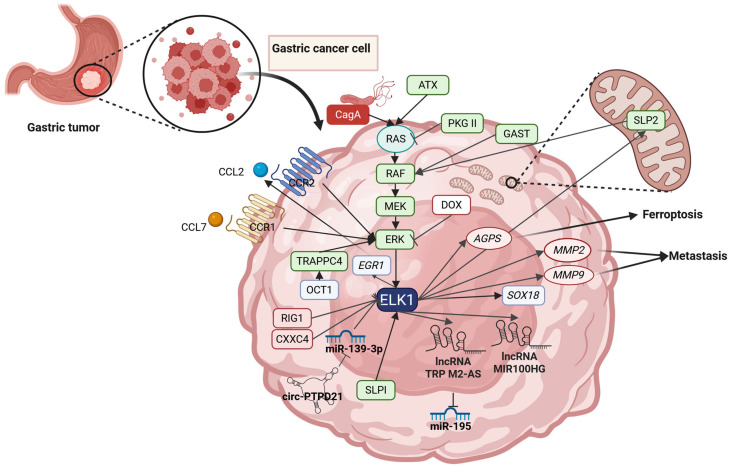
ELK1 in Gastric Cancer. This figure summarizes key pathways in which ELK1 is implicated in Gastric cancer (GC). Pointy solid-line arrows stand for direct activation while arrows with fading-tails represent indirect activation. Blunt solid-line arrows stand for direct inhibition while blunt arrows with fading tails stand for indirect inhibition. Carcinogenic/oncogenic substances are depicted red and anticancer agents light blue. Created in BioRender. Kalampounias, G. (2025) https://BioRender.com/9ltkpow.

ELK1 has been reported multiple times in the context of Gastric cancer (GC). For the first time in 1997, ELK1 was identified as a target of Gastrin (GAST), via the Raf-MEK-MAPK pathway activation by the gastrin receptor [179] (Figure 1). The activation of ELK1 was correlated to pro-tumorigenic phenomena [179]. *H. pylori*, harboring the Cytotoxin-associated gene A (CagA) protein, was later identified as another MAPK activator which leads to ELK1 activation (together with c-Fos and c-Jun), ultimately leading to GC tumorigenesis [180,181,182]. In Nishigaki et al., 2005, *ELK1* was identified as a gene silenced in normal stomach mucosal tissues; whereas, it is epigenetically activated in gastric cancer specimens [183]. Moreover, in a 2014 study, the *ELK1* gene was listed among a set of hypomethylated genes often reported in metastatic GC, underscoring its role in GC pathophysiology and its potential role as a biomarker [184].

In gastric cancer, ELK1 has also been credited with a pivotal role in Hedgehog-EMT signaling crosstalk [185]. ELK1 gene was identified as a downstream component Hedgehog (Hh) signaling, being a GLI1 target [185]. GLI1 knock-down reduced *ELK1*’s expression, while *ELK1* knockdown led to a decrease in GC cell growth [185]. ELK1 was found to be implicated in the Secretory leukocyte protease inhibitor (SLPI) signaling [186]. SLPI is overexpressed in GC, contributing to migration through degradation of the Extracellular matrix (ECM) [186]. ELK1 was identified as a downstream component of the SLPI cascade, since SLPI knockdown using siRNA diminished ELK1 phosphorylation [186]. ELK1 was also shown to regulate the expression of the Matrix metallopeptidase 2 (MMP-2) and Matrix metallopeptidase 9 (MMP-9) proteins, contributing to GC cell migration [186]. The role of ELK1 in cancer cell aggressiveness was also validated by another study focusing on Octamer transcription factor 1 (OCT1; also known as POU domain, class 2, transcription factor 1, POU2F1) [187]. Qian et al. reported that OCT1 transactivates synbindin (officially known as Trafficking protein particle complex subunit, TRAPPC4) and finally leads to ERK1/2 activation [187]. This targets the ELK1 and Ribosomal S6 kinases (RSKs) TFs, ultimately enhancing proliferation and migration rate [187]. Mitochondrial activity is also partially regulated by ELK1 as indicated by a positive feedback mechanism involving Stomatin-like protein 2 (SLP2) and MAPK-ELK1 signaling [188]. In GC, SLP2 overexpression was found to stimulate RAF1 and ultimately lead to ELK1 activation, whereas loss of SLP2 significantly suppress ELK1 phosphorylation [188]. On the other hand, activated ELK1 was found to bind the SLP2 promoter and initiate its transcription, highlighting the positive feedback loop connecting the two proteins [188]. This study was the first identifying a direct interaction between the mitochondrion and ELK1, indicating the multifaceted roles of ELK1 in tumorigenic phenomena. ELK1’s role in GC C-C motif chemokine ligand 7 (CCL7)-mediated metastasis was also reported in a 2020 study by Chen et al. [189]. The TF SRY-box transcription factor 18 (SOX18) was found elevated in metastatic GC patients and its activation mechanism involved activation of the CCL7-CCR1 (C-C motif chemokine receptor 1) pathway [189]. The activation process was found to rely on successful MAPK-ELK1 activation [189]. *ELK1* silencing results in reduced SOX18 expression, independently from CCL7 stimulation, indicating the role of ELK1 in SOX18 regulation [189].

Another form of ELK1 involvement in pro-tumorigenic activity is by regulating the expression of noncoding RNAs with pro-survival and pro-proliferative activity [38,39,190]. One of these molecules is lncRNA TRPM2-AS which is regulated by ELK1 and whose overexpression was correlated to increased invasion and metastatic potential in GC cell lines and tumor tissues [38]. TRPM2-AS acts as a sponge of miR-195, which has a known tumor-suppressor role [38]. The role of ELK1 in long noncoding RNA regulation was also highlighted by another study in GC, focusing on the lncRNA MIR100HG [39]. MIR100HG is a known oncogene in several cancer types and was found to have ELK1 binding sites on its promoter [39]. The same study investigated how Cysteine-X-X-Cysteine (CXXC) proteins affect ELK1 phosphorylation and concluded that CXXC finger protein 4 (CXXC4) overexpression in GC cells can successfully suppress ELK1 phosphorylation [39]. Finally, the study reports that CXXC4 overexpression inhibits ELK1 translocation inside the nucleus thus rendering ELK1 impotent [39]. The circular RNA Circ-PTPDC1 is another example of ELK1’s role in tumorigenesis [190]. Circ-PTPDC1 acts as sponge of the tumor suppressor miR-139-3p, which is a micro-RNA targeting *ELK1* [190]. The study confirmed that miR-139-3p can decrease ELK1 protein levels by halting its expression, while knocking-down circ-PTPDC1, further diminishes ELK1 presence [190].

ELK1 downregulation was shown to be a target of the tumor suppressor gene Retinoic acid-inducible gene I (*RIGI*) [191]. RIG-I-MYC and RIG-I-EGFP fusion proteins reduced the levels of ELK1 and c-Jun and according to Huang et al. 2002, cytostatic and apoptotic effects were observed [191]. Additionally, cGMP-dependent protein kinase 2 (PKG2), was shown to inhibit proliferation of GC cells by suppressing MAPK signaling [192]. Mechanistically it was shown that PKG2 overexpression downregulated ELK1 activation via the inhibition of RAS-RAF-MEK-MAPK signal transduction, underscoring ELK1’s role in GC cell survival [192]. In another study, ELK1 was identified as a downstream target of doxycycline, after treatment of GC cells, since the antibiotic was found to significantly decrease ERK1/2 phosphorylation [193] (Table 4).

**Table 4:** Pharmaceuticals targeting ELK1 in gastric, head, neck, nasopharyngeal, liver, and cervical cancer.

Cancer	Substance	Model/Sample	Effect(s) on ELK1	Mechanism	Outcome	Ref.
GC	Doxycycline	Human GC cell lines (AGS, MKN-45 and KATO III)	Decrease in ELK1 phosphorylation	Inhibition of RAS-RAF-MEK-MAPK-ELK1 signaling	Growth inhibition	[193]
GC	Grifolin	Human GC cell line (MGC803)	Decrease in ELK1 phosphorylation	Direct inhibition of ERK1/2 activity	Decrease in cell adhesion, migration and invasion	[104]
HNSCC	Baicalein	Human OSCC cell lines (SCC-4 and CAL-27)	Decrease in ELK1 phosphorylation	Inhibition of RAS-RAF-MEK-MAPK-ELK1 signaling	Reduced proliferation and migration	[194]
HNSCC	Mebendazole (MBZ)	Human HNSCC lines (SCC-15 and CAL27)	Decrease in ELK1 phosphorylation	Modulation of cancer-associated pathways including ELK1/SRF, AP-1, STAT1/2, MYC/MAX	Decrease in cell cycle progression, proliferation, and migration	[178]
NPC	Grifolin	Human NPC cell lines (CNE1 and 5–8F) and mice xenografts	Decrease in ELK1 phosphorylation	Direct inhibition of ERK1/2 activity	Decrease in cell adhesion, migration and invasion	[104]
HCC	(JS-K)	Human HCC cell lines (HepG2 and Bel-7402)and mice xenografts	Decrease in ELK1 phosphorylation	Activation of JNK and p38 MAPK and inactivation of Raf/MEK/ERK signaling pathways	Apoptosis induction	[100]
HCC	TD52	Human HCC cell lines (Sk-Hep1, PLC5, Hep3B and Huh-7)	Decrease in ELK1 phosphorylation	Inhibition of CIP2A and promotion of PP2A expression	Apoptosis induction	[53]
HCC	Withaferin A (WFA)	Mice models, xenografts, and human HCC cell lines (HepG2 and Huh7)	Increase in ELK1 phosphorylation	Crosstalk between ERK/RSK, ELK1, and DR5	Apoptosis induction	[195]
HCC	2-(2-mercaptoethanol)-3-methyl-1,4-naphthoquinone (Compound 5, Cpd 5) plus EGF	Human HCC cell line (Hep3B)	Increase in ELK1 phosphorylation	Prolonged MAPK phosphorylation	Growth inhibition	[196]
CC	Luteolin	Human CC cell line (HeLa)	Decrease in ELK1 expression	Decreases in the expression of several pro-survival genes including *ELK1*, *MAPK14*, *MAP3K5*, *MAPK3* and *MAPK1*	Proliferation inhibition, apoptosis induction	[197]
CC	*Kaempferia parviflora* plant extract	Human CC cell line (HeLa)	Decrease in ELK1 phosphorylation	Inhibition of MAPK and PI3K-Akt signaling	Apoptosis induction	[198]
CC	Tanshinone I	Human CC cell lines (HeLa and C4-1)	Decrease in ELK1 phosphorylation	Downregulation of KRAS expression	Inhibition of metastasis and cisplatin resistance	[199]
CC	Grifolin	Human CC cell line (HeLa)	Decrease in ELK1 phosphorylation	Direct inhibition of ERK1/2 activity	Decrease in cell adhesion, migration and invasion	[104]

Abbreviations: GC = Gastric cancer; HNSCC = Head and neck squamous cell carcinoma; NPC = Nasopharyngeal carcinoma; HCC = Hepatocellular carcinoma; CC = Cervical cancer.

Regarding drug resistance, ELK1 has been reported as a key contributor of cisplatin resistance, by forming a positive feedback loop with C-C motif chemokine ligand 2 (CCL2; also known as Monocyte chemoattractant protein-1, MCP-1) and maintaining EMT characteristics in resistant cells [177]. CCL2 was found overexpressed as a result of EGR1, an overexpression that leads to ELK1 activation via the ERK1/2 pathway [177]. Since it is also known that ELK1 actively participates in *EGR1* expression, the positive feedback loop identified sheds light in a potential drug resistance pathway. Another activator of ELK1 has been identified to be Autotaxin (ATX), also known as ectonucleotide pyrophosphatase/phosphodiesterase 2 (ENPP2), which has been found to assist GC cells evade paclitaxel-induced apoptosis [200]. ATX was found to induce the expression of Osteopontin (OPN) as well as MAPK signaling [200]. Both proteins were found to be crucial for pro-survival activity, mediated by ELK1, indicating an axis assisting in resistance against paclitaxel [200]. Finally, a recent study identified a role for ELK1 in apatinib (also known as rivoceranib) resistance [201]. Wang et al. in 2025, identified ferroptosis tolerance as an apatinib-resistance mechanism and correlated it with decreased polyunsaturated ether phospholipid synthesis, which was a result of enzyme downregulation [201]. Alkylglycerone phosphate synthase (AGPS) a major enzyme in the pathway, the transcription of which is regulated by ELK1, was found downregulated, as was the case for ELK1 [201].

### 2.6. Esophageal, Head, Neck, and Laryngeal Cancers

#### 2.6.1. Esophageal Adenocarcinoma

ELK1 was reported to be upregulated in Esophageal cancer (ESCA) cells and was correlated with poor prognosis, in a recent study by Tang, Sun et Cai [202]. Its upregulation was reported to positively affect the expression of Peroxiredoxin 4 (PRDX4) thus promoting cancer progression by acting as a cytoprotective mechanism against oxidative-stress-induced damage [202]. Moreover, the study presented significant mechanistic details about ELK’s regulation. ELK1 was found to be the target of the tumor-suppressor miR-29a-3p, which negatively regulated ELK1’s expression [202]. Additionally, the lncRNA THUMPD3-AS1 was found to sponge miR-29a-3p, forming a transcriptional loop that promotes proliferation, migration and invasion of ESCA cells [202].

#### 2.6.2. Esophageal Squamous Cell Carcinoma

ELK1 was first reported in Esophageal squamous cell carcinoma (ESCC) in 2006, in a study by Chen et al. In that study, in 84 out of 107 analyzed tissue samples, ELK1 was found to be overexpressed [203]. However, in another study, in which a limited number of tissue samples was used (eight), ELK1 was reported as a downregulated TF in ESCC [204]. In ESCC was shown that MEK inhibitors, targeting the RAS-RAF-MEK-MAPK-ELK1 pathway, activate STAT3-mediated signaling, while dual inhibition of the two pathways resulted in decreased cell proliferation [205]. The connecting link was identified to be the *SOCS3* gene and its promoter [205]. SOCS3, a known suppressor of the JAK/STAT pathway was identified as a downstream target of ELK1, which promotes its expression, thus silencing STAT signaling [205]. Upon MEK inhibition, STAT3-dependent transcription was found to be activated, acting as a rescue mechanism in ESCC [205].

#### 2.6.3. Head and Neck Squamous Cell Carcinoma

A study on Head and neck squamous cell carcinoma (HNSCC) on rodent models revealed that Protein phosphatase 6 deficiency (Ppp6c or PP6) leads to increased Ras signaling which induces ELK1 activation [206]. PP6 has been found to limit the growth of KRAS- and Braf-mutant cancer models thus exhibiting tumor-suppressing activity [206]. ELK1 has also been identified in Oral squamous cell carcinoma (OSCC) cell lines. Treatment of OSCC cells with the flavonoid baicalein, a known tumoricidal agent, caused significant reductions in ERK1/2 and ELK1 phosphorylation and suppression of the cells’ proliferative, migratory, and metastatic potential [194]. Another study focused on an anthelminthic drug, mebendazole as a potential anti-HNSCC treatment [178]. Mebendazole (MBZ) was found to suppress cell cycle progression, proliferation, and migration, while it also affected ELK1 signaling [178]. ELK1’s expression was increased both in a dose-dependent and a time-dependent manner [178]. Lastly, the antiangiogenic protein Insulin-like Growth Factor-Binding Protein 3 (IGFBP-3) was also tested on HNSCC and was found to significantly reduce ERK1/2 and ELK1 activation [47]. The study also reported a significant downregulation of EGR1 following treatment with IGFBP-3, which was a result of reduced promoter activity, due to ELK1 inhibition and loss of subsequent SRE-ELK1-mediated transcription of the *EGR1* gene [47]. Finally, in a recent study, the infiltration of HNSCC by monocytes and macrophages was studied as a potential diagnostic/prognostic factor [207]. In monocytes from the peripheral blood, Bestrophin 1 (BEST1) was found elevated which was attributed to tumor-excreted Vascular endothelial growth factor A (VEGF-A) signaling [207].VEGF-A activates the RAS-RAF-MEK-ERK cascade, and as a result, ELK1 is activated leading to the transcription of the *BEST1* gene [207]. The study emphasized the use of BEST1 expression as HNSCC marker, since it was overexpressed only in tumor-associated mononuclear cells [207].

#### 2.6.4. Laryngeal Squamous Cell Carcinoma

In laryngeal squamous cell carcinoma (LSCC), miR-340-3p was identified as suppressor of ELK1, inhibiting its transcription by binding near on *ELK1* mRNA [208]. Overexpression of miR-340-3p leads to tumor suppression, while mir-340-3p knocking-down leads to ELK1 overexpression and elevated proliferation, migration, colony formation, and invasion [208]. The same study also identified the circular RNA circPPFIA1 as a miR-340-3p suppressor. CircPPFIA1 was found to be overexpressed in LSCC, while its knocking-down significantly impairs the cells’ ability to proliferate and migrate [208].

#### 2.6.5. Epidermoid Squamous Cell Carcinoma

Using the A431 cell line, another study focused on potential roles of the Inhibitor of DNA binding 3 protein (ID3) in squamous carcinoma cells [209]. ID3 has contradicting roles ranging from synergy with oncogenes to inducer of apoptosis. In this study, by Chen et al. in 2015, ID3 was found to elevate ELK1 levels; however, the result was decreased viability [209]. The analysis of apoptotic factors revealed that ELK1 caused procaspase-8 overexpression [209]. Inversely, ELK1 knockdown reduced both active caspase-8 and its precursor, validating the ELK1-dependent apoptosis [209].

#### 2.6.6. Nasopharyngeal Carcinoma

Up to this day, only two studies have been conducted on Nasopharyngeal carcinoma (NPC) about ELK1’s role in disease progression [104,210]. A 2015 study by Luo et al. reported that Grifolin, a secondary metabolite of the mushroom *Albatrellus confluens* can decrease the phosphorylation of ELK1 by directly biding to ERK1/2 and thus inhibiting their kinase activity [104]. Grifolin suppressed cell adhesion, migration and invasion of human NPC (CNE1 and 5–8F), GC (MGC803), BC (MCF-7 and MDA-MB-231) cells and cervical cancer (HeLa) cells. Additionally, it exhibited anti-metastatic potential in metastatic mouse xenografts of 5-8F NPC cells [104]. Zhao et al. in 2020, identified PCNA clamp associated factor protein (PCLAF; formerly known as KIAA0101), as a significant contributor to tumorigenesis [210]. PCLAF was found to be highly expressed in NPC, and its knocking-down leads to cell proliferation suppression and apoptotic activity induction. ELK1 was identified as a regulator of *PCLAF* expression, since eight sites for ELK1 binding were found on its promoter. ELK1 was also overexpressed in tumors high in PCLAF, whereas ELK1 knockdown significantly downregulates the expression of the protein [210].

### 2.7. Liver Cancer

Over the years, a significant number of studies on ELK1 in liver (or hepatic) cancer have been conducted. The vast majority of data is about Hepatocellular Carcinoma (HCC) which is the most common malignancy originating in the liver. Several studies have investigated how ELK1 can stimulate the expression and activation of other TFs, and especially EGR1, which is a known pro-oncogenic factor. In 1999 it was shown thar hypoxia stimulated EGR1 activation via activation of the SRF and ELK1 TFs by the Raf-MEK-MAPK pathway [211]. In 2019, Shan et al. studied how ER stress in HCC can induce EGR1 expression and activation and concluded that Src activates the RAS-RAF-MEK-MAPK pathway which phosphorylates and stabilizes the TF ELK1 [212]. ELK1 and SRF were then reported to induce EGR1 gene transcription; however, ELK1 silencing can have differential effects on EGR1 expression [212]. The authors report that ELK1 knockdown does not downregulate baseline EGR1 levels; however, the cell’s ability to induce EGR1 expression during ER stress is lost in the absence of ELK1 [212]. On the other hand, SRF knockdown downregulates EGR1 mRNA levels, but plays a minor role in ER-stress-related induction of EGR1’s transcription [212]. The study underscored the importance of ELK1-SRF interaction in the expression of EGR1, highlighting the fact that each TF contributes differently regarding gene expression upon stimulation by stress conditions [212]. These findings built upon previous data regarding EGR1 expression, in which the activation of ELK1 is required for its expression in HCC cells, following stimulation by the known carcinogen 12-O-tetradecanoylphorbol-13-acetate (TPA) [213]. ELK1-mediated EGR1 activation has also been reported as a result of amino acid limitation in HCC cells [214]. The study reported that EGR1 activation was a result of MEK-MAPK-Elk1 activation which led to transcription of the EGR1 gene, instead of the known Eukaryotic translation initiation factor 2-alpha kinase 4 (EIF2AK4 or GCN2)—Activating transcription factor 4 (ATF4) pathway (GCN2-ATF4).

Besides EGR1, ELK1 has been reported to promote the activation of several other TFs, all credited with important roles— in various aspects of tumorigenesis, tumor progression and aggressivity. Another study by Shan et al. in 2015, identified ELK1 as the TF mediating *FOS* gene transcription, upon stimulation of the RAS-RAF-MEK-MAPK signaling cascade [215]. As an initial stimulus, Amino acid deprivation (AAD) was studied which causes significant induction of stress-related genes [215]. Phosphorylation of ELK1 occurred after AAR and resulted in c-Fos elevated expression, providing insight about a stress mechanism involving ELK1 in HCC cells [215]. Another study reported thar resveratrol treatment of HCC cells, elevates ELK1 and c-Jun’s activation, thus promoting AP-1 activity [216]. ELK1’s role in c-Jun expression was also studied by Zhong et al. in 2007 [217]. Elk1 was found to regulate the expression of TATA-binding protein (TBP), which is a transcription initiation factor [217]. TBP is a crucial part of c-Jun’s expression, thus indicating a correlation between ELK1’s activation and c-Jun’s expression [217]. The study reports that the activity of the JNKs were shown to differentially affect ELK1 activation, with JNK1 increasing its phosphorylation, while JNK2 decreasing it [217]. Given that JNKs main target is c-Jun, the study elucidated a mechanism describing how JNK1 forms a positive feedback loop of cJun activation while JNK2 participates in a feedback inhibition loop [217]. ELK1 was also implicated in the expression of One Cut homeobox 2 (ONECUT2) In HCC cells [218]. ONECUT2 is reported to act as an oncogene, regulating the expression of Fibroblast growth factor 2 (*FGF2*) and ATP citrate lyase (*ACLY*) [218]. FGF2 and ONECUT2 were also reported to participate a positive feedback loop in which the connecting link is ELK1 [218]. FGF2 signaling via the RAS-RAF-MEK-ERK pathways was found to activate ELK1, the TF responsible for ONECUT2 expression [218]. In a recent study in HCC, ELK1 was found to regulate the expression of *FOXC1*, a significant contributor in metabolism regulation, migration, and invasion [219]. FOXC1 was found to be overexpressed in HCC cells as a result of ERK1/2 activation by ROS [219]. The activation of ELK1 was found to be crucial in FOXC1 expression which were both reported to be positively correlated [219]. Through FOXC1, ELK1 was also found to promote DNA hypermethylation of the promoter of the Cystathionine γ-lyase (CTH) gene, thus playing a significant role in the regulation of cysteine metabolism [219]. CTH and FOXC1 were shown to have conflicting roles regarding migration, since FOXC1 exhibited pro-migratory activity, while CTH reduced the cells’ metastatic potential [219].

Several studies have also explored the role of ELK1 in intracellular signaling regulation through the expression of receptor ligands, scaffolding proteins, and apoptosis regulators. In a 2020 study, a correlation between Insulin-like growth factor binding protein-2 (IGFBP2) and ELK1 was observed [220]. Moreover, elevated IGFBP2 has been observed in multiple cancers and the authors reported that in HCC patients its levels are risen as well [220]. The study reports that HCC cells treated with IGFBP2 have also elevated proliferation and cell adhesion signaling activation (phosphorylation of Focal Adhesion Kinase was found elevated) [220]. As a downstream target of IGFBP2, ELK1 was identified, which was found activated via ERK1/2 activation [220]. Another mechanism involving ELK1 and growth factor signaling was published in 200 and focused on loss/silencing of Insulin-like growth factor binding protein 3 (IGFBP-3) [221]. In HCC, IGF-I was reported to exhibit significant mitogenic activity, which could be reversed by IGFBP-3 [221]. IGF-I was found to induce the phosphorylation of ELK1 in HCC cells [221], indicating that ELK1 targeting could attenuate growth-factor-related mitogenic signaling. A recent study identified SRF, ELK1, and ELK4, as the TFs promoting the expression of Minute homolog 4 (MDM4), a known TP53 inhibitor, in HCC cells [222]. Additionally, ELK1 overexpression caused increases in MDM4 expression [222]; whereas, silencing ELK1 and knocking down SRF significantly reduced MDM4 expression levels [222]. These observations shed light to a significant SRF-ELK1 (and SRF-ELK4) interaction which drives TP53 suppression and promotes cell cycle progression. Another study investigated how ELK1 overexpression in HCC tissues is connected the expression levels of Sperm-associated antigen 9 (SPAG9) [223]. SPAG9 was found to be correlated to metastatic potential by promoting migration and migration, while its knocking down was demonstrated to downregulate both functions [223]. SPAG knockdown also affected ELK1 expression as well as p38 MAPK signaling [223]. The authors report that ELK1 overexpression overthrows SPAG9’s silencing effects, while silencing the *ELK1* gene leads to inhibition of HCC migration activity [223].

ELK1 has been credited with multiple roles regarding EMT, being a connecting link of molecular networks that underly the phenomenon. A study by Zhang et al. (2022) identified the role of ELK1 as a regulator of ETS variant transcription factor 1 (ETV1) expression levels in human HCC specimens, upon activation by Hepatocyte growth factor (HGF) [224]. Both TFs, ELK1 and ETV1 belong to the ETS family and ETV1 was found to be elevated in HCC patients, being a poor prognosis factor and a metastasis driver by upregulating the expression of Protein-tyrosine kinase 2 (PTK2; also known as Focal adhesion kinase 1, FAK1) and MET proto-oncogene (MET; also known as Hepatocyte growth factor receptor, HGFR) [224]. Inhibition of both kinases was shown to reverse the pro-metastatic effects of ETV1 in HCC cells, while ETV knockdown cells were not stimulated effectively by HGF [224]. ETV1’s expression was shown to be the target of ELK1 activation via the RAS-RAF-MEK-ERK1/2 pathway, following stimulation with HGF, and ERK1/2 was found to inhibit its expression [224]. Another study regarding ELK1’s role in EMT revealed that the transcription co-activator p300 can regulate the expression of the atypical Protein kinase C iota (PRKCI or aPKC-ι), indirectly, via the activation and stabilization of ELK1 [225]. The same group had established the role of aPKC-ι in the EMT process in HCC [226]; however, the current study elucidated how ELK1 signaling is a mediator of this process. The combination of p300 activation with the signals received by ELK1 from its main upstream activators (ERK1/2), seems to promote the expression of aPKC-ι, shedding light to significant EMT drivers [225]. Besides aPKC-ι, ELK1 has also been reported to control PKCα (PRKCA) expression [227,228,229]. Evidence for this mechanism were first reported in HCC in 2005, in a study in which ELK1 and MZF1 antisense oligonucleotides were reported to reduce *PRKCA* mRNA levels and significantly impair cell proliferation, migration, and invasion [229]. A later study (2015), by Yue et al., confirmed that in HCC, the expression of PKCα is regulated by ELK1 and MZF1, which both interact, directly bind and activate transcription on the PKCα gene promoter, a finding which was confirmed by later studies as well [227,228]. The study also describes how DNA-binding-deficient (∆DBD) forms of ELK1 (ELK1∆DBD) and MZF1 (MZF1∆DBD) fail to increase PKCα expression, regardless of their overexpression by plasmid vectors, while in experiments of functional ELK1 and MZF1 overexpression, PKCα is upregulated [227]. PKCα deficiency was correlated to decreased proliferation, migration, and invasion, whereas upregulated PKCα leads to increased tumorigenicity and cell growth in HCC [227]. Another study reported again that MZF1/ELK1 expression is correlated with that of PKCα in HCC; however, they did not confirm this in BCa or lung cancer [228]. They concluded that ELK1-MZF1 artificial dissociation decreases PKCα expression and thus suppresses EMT-related characteristics [228]. In a 2000 study, ELK1 was found to be a downstream target of Sphingosine 1-phosphate (S1P), which promotes proliferation and migration of HCC cells [230]. From a mechanistic point of view ELK1 and SRF were activated following phosphorylation of ERK1/2 [230]. The MAPK pathway was found to be a downstream target of the G protein-coupled receptors Lysophosphatidic acid receptor 3 (LPA3; also known as EDG3) and Sphingosine-1-phosphate receptor 2 (S1PR2; also known as EDG5), ultimately leading to the activation of c-JUN and c-FOS [230].

Regarding ELK1 interactions with noncoding RNA molecules, several mechanisms have been identified in HCC. In 2025, Heo et al., identified miR-361-3p as a suppressor of ELK1’s expression in HCC cell lines and tissues [21]. miR-361-3p was also identified as a target of the circular RNA circMFN2, which was found to act as a sponge [21]. circMFN2 overexpression was found to elevate cell proliferation, migration, and invasion, whereas its silencing led to decreased cell growth [21]. ELK1 also acts as regulator of circMFN2 expression, since its binding to the MFN2 promoter further enhances its expression [21]. Thus, a positive feedback loop was discovered [21]. Additionally, the study discussed how ELK1 is also implicated in glutaminolysis, suggesting its role in the metabolic adaptation of HCC cells that further drives cell growth [21]. In a 2023 study by Su et al., the ERK/ELK1/Snail axis was found to be the target of an alternative splicing mechanisms involving the long noncoding RNA LINC01089 in HCC cells [231]. LINC01089 was found to interact with the Heterogeneous nuclear ribonucleoprotein M (hnRNPM) and induce the skipping of Diaphanous-related formin-3 (DIAPH2) exon 3 [231]. The authors report that upon LINC01089 silencing this does not occur and the overexpressed DIAPH3 protein suppressed ELK1’s activation [231]. Therefore, the study elucidates a link between LINC01089 expression and ELK1 activity, suggesting targeting the lncRNA or exploring its prognostic value [231].

Targeting ELK1 directly or indirectly is another major strategy in HCC, since ELK1 has been identified a major driver of tumorigenesis and tumor growth. 2018 study by Ranjan et al. investigated the mechanism of action of MDM2 Binding Protein (MTBP) on HCC cells [232]. Given that in a previous study they highlighted MTBP as a suppressor HCC [233], the mechanistic analysis revealed that MTBP targets ERK1/2 and reduces ELK1-mediated transcription [232]. Since ELK1 is a downstream component of the RAS-RAF-MEK-ERK1/2 pathway, translocation of activated ERK inside the nucleus is needed to facilitate ELK1’s activation and promote ELK1-mediated gene transcription [232]. MTPB was shown to inhibit the interaction between p-ERK1/2 and Importin-7 (IPO7 or RanBP7), an importin that translocated ERK inside the nucleus [232]. MTBP increased the cytoplasmic fraction of p-ERK1/2 leading to reduction in migration and invasion of HCC cells [232]. This study revealed an important aspect of a potential ELK1 targeting approach, underscoring the role of importins in the regulation of ELK1’s activity. Another study investigated how exogenous Nitric Oxide (NO) can induce apoptosis on HCC cells [100]. The study reported that the administration of O2-(2,4-Dinitrophenyl) 1-[(4-ethoxycarbonyl)piperazin-1-yl]diazen-1-ium-1,2-diolate (JS-K) which is an exogenous NO donor able to release NO following activation by Glutathione S-transferases (GSTs), can reduce MAPK-mediated signaling by inhibiting c-Raf activation [100]. The study identified ELK1 as one of the molecular targets of JS-K, the activation of which was reduced and thus the subsequent transcription activity [100]. A 2011 study investigated how Caveolin-2 (CAV2) can affect cell proliferation and intracellular signaling in various cancer types [234]. CAV2 transfection in HepG2 HCC cells caused a significant reduction in cell proliferation and growth, as well as a delayed entry in the S phase [234]. Mechanistically, it was shown that CAV2-transfected cells had decreased ELK1 and STAT3 activation, explaining the impairment of proliferation and viability [234]. A 2014 study by Yu et al. showed that TD52, an erlotinib derivative can effectively induce the apoptotic death of HCC cells and reactivate PP2A [53]. The study reports that TD52 can affect the binding of ELK1 to the proximal promoter of the CIP2A gene, thus downregulating transcription of CIP2A [53]. CIP2A has also been found to regulate PP2A expression [50,53], and given the fact PP2A was shown to affect bortezomib and erlotinib cytotoxicity in HCC cells [52], its reactivation could be a significant component against drug resistance. Since CIP2A has also been reported as an ELK1 target in ovarian, uterine cancer, and liver cancer [235], its inhibition by erlotinib provided a new mechanistic approach which could be applicable in more cancer types. A 2008 study by Ying et al. demonstrated how HCC cells treated with ELK1 antisense oligonucleotide (ODN) exhibit decreased viability and tumor growth [236]. Following treatment with ELK1 ODN, the levels of PKCα were also found to be decreased, indicating reduced pro-oncogenic signal transduction and a less aggressive phenotype [236].

Moreover, it is noteworthy that there are some studies reporting that activated ELK1 can also contribute to HCC suppression. In a 2017 study conducted on mice, ELK1 was credited with a contradictory role regarding HCC tumorigenesis [195]. Kuppusamy et al. investigated whether Withaferin A (WFA), a bioactive molecule derived from *Withania somnifera*, can exhibit cytotoxicity against HCC and several signaling cascades were assessed with phosphokinase panels [195]. Elk1 was found among a set of phosphorylated genes in WFA responses and the authors suggested that crosstalk between ERK/RSK, ELK1, and DR5 could be an important target of HCC inhibition [195]. A 2002 study conducted on hepatoma cells, concluded that prolonged ELK1 phosphorylation can inhibit the growth of the cells [196]. Hep3B cells were treated simultaneously with EGF and 2-(2-mercaptoethanol)-3-methyl-1,4-naphthoquinone (Compound 5, Cpd 5), which is a protein-tyrosine phosphatase (PTPase) inhibitor [196]. The results indicated that although ERK1/2 and ELK1 were found to be activated, the prolonged phosphorylation in fact suppressed cell growth [196].

ELK1 has also been reported in the context of oxaliplatin (OXA) resistance in HCC [237]. A 2017 study by Ma et al., reports that Bone morphogenetic protein-4 (BMP4) which is a driver of EMT is also overexpressed in OXA-resistant HCC tissues [237]. Targeting BMP4 was identified as a way to resensitize cells to OXA, and thus, the underlying mechanisms of BMP4 activity were studied [237]. ELK1 was found to be overexpressed in OXA-resistant cells, and its expression and activation were both found to be BMP4-depedent, via activation of MEK1 and ERK1/2 [237]. Inhibition of the MAPKs using PD98059 or ELK1 silencing led to cell resensitization, indication the pivotal role ELK1 has in chemoresistance [237].

### 2.8. Thyroid Cancer

**Figure 2:**
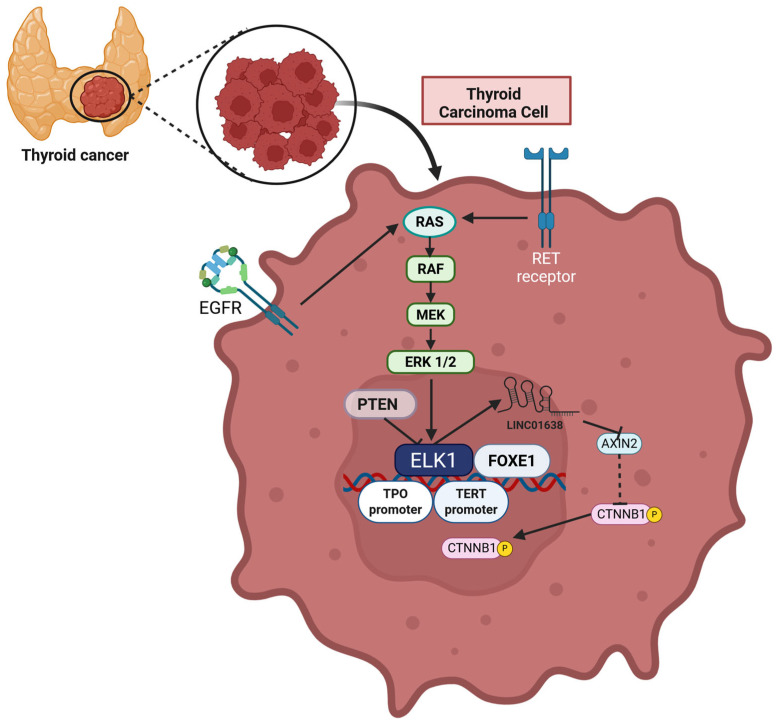
ELK1 in Thyroid Cancer This figure summarizes key pathways in which ELK1 is implicated in Thyroid cancer (TC). Pointy solid-line arrows stand for direct activation while arrows with fading-tails represent indirect activation. Blunt solid-line arrows stand for direct inhibition while blunt arrows with fading tails stand for indirect inhibition. Dashed arrows indicate the loss of the depicted action. Created in BioRender. Kalampounias, G. (2025) https://BioRender.com/p2k18dz.

In Thyroid cancer (TC), data remain relatively scarce. A 2009 bioinformatics study focused on Death-associated protein 3 (DAP3) expression in TC tumors, revealed that ELK1 is overexpressed (compared to non-cancerous tissues) and could be a regulator of DAP3 expression [238]. The authors highlighted the importance of DAP3, since previous studies have shown that its overexpression was correlated to advanced disease stage on gliomas and thymomas [239,240]. Another study revealed that the TFs ELK1 and Forkhead box protein E1 (FOXE1) interact, and this interaction may be associated with thyroid cancer risk [241] (Figure 2). ELK1 was found to recruit FOXE1 to the Telomerase reverse transcriptase (*TERT*) and the Thyroid Peroxidase (*TPO*) promoters, while disruptions of ELK1 activation using MEK inhibitors were found to inhibit ELK1-FOXE1 interactions [241]. A significant observation about ELK1 was that its suppression causes an induction of EGR1 and Phosphatase and tensin homolog (PTEN) and leads to cytostatic and pro-apoptotic effects [242]. Moreover, the study confirmed previous findings regarding ELK1’s upregulation in TC compared to normal tissues [242].

Finally, a more recent study by Lv and Xue in 2021 reported a mechanism involving ELK1 in Papillary thyroid carcinoma (PTC) progression [40]. ELK1 was proposed as a TF of the known oncogenic lncRNA LINC01638, which has been found to be upregulated in PTC cells [40]. ELK1 overexpression leads to LINC01638 upregulation, while ELK1-knockdown cells have decreased LINC01638 levels [40]. LINC01638 was found to regulate cell cycle progression, proliferation, migration, and invasion, while it also affects intracellular signaling cascades [40]. Knocking down of LINC01638 leads to MYC reduction and increases in Axis inhibition protein 2 (AXIN2) levels [40]. The inhibition of AXIN2 by LINC01638 seems to be vital in Wnt/β-catenin signaling regulation since upregulations in the AXIN2 levels reduce β-catenin and Cyclin D1 [40]. The study underscores the role of ELK1 as a Wnt signaling regulator, indicating its pivotal role in pro-proliferation and pro-migration pathways [40].

### 2.9. Cervical Cancer

In cervical cancer (CC), several recent studies have explored the role ELK1 in tumorigenesis and progression, especially through the scope of RNA regulation. Additionally, due to HeLa cells being one of the most studied and old in vitro pan-cancer models, amplitude of data exists regarding signaling cascades and transcription regulation mechanisms. In a 2020 study by Zhang and Zhang, the connection of the circular RNA circRNA_0000285, the micro-RNA miR-197-3p, and ELK1 was investigated [243]. miR-197-3p was shown to target *ELK1* and significantly impair cell cycle progression and viability of CC cells via apoptosis and autophagy activation [243]. Circ_0000285 was able to suppress miR-197-3p activity by acting as a sponge and was found to be overexpressed in CC tissues and cells [243]. Knocking down circ_0000285 directly affected cell cycle progression and viability both in vitro and in vivo [243]. On the other hand, miR-197-3p knockdown did not inhibit cells with a silenced circ_0000285, indicating that the detrimental effects of circ_0000285 silencing were miR-197-3p-dependent [243]. Since ELK1 was identified as a direct target of miR-197-3p, the TF’s role in CC development was theorized to be crucial. Moreover, the circular RNA hsa_circ_0000515 has been shown to be a positive regulator of ELK1 expression [88]. Hsa_circ_0000515 was found to sponge miR-326 and thus increases in the circular RNA levels lead to increased tumor progression by ELK1 expression [88]. Since miR-326 has been identified as an ELK1 silencer [244], this study sheds light to a novel axis including miR-326/hsa_circ_000515/ELK1 [88]. On the contrary, silencing of hsa_circ_0000515 and/or miR-326 upregulation suppression tumor growth and inhibit invasion [88]. The pro-tumor activity of hsa_circ_0000515 through ELK1 was evident in both CC tissues and cells, underscoring the importance of the circular-RNA as a potential therapeutic target, as well as that of miR-326 as a treatment strategy [88,244]. Another study identified the relation between ELK1 and miR-130b-5p [245]. ELK1 and miR-130b-5p were found to be negatively correlated, as the overexpression of one of them suppresses the other [245]. In CC cells, decreased miR-130b-5p is reported while ELK1 is found upregulated [245] (Table 5). On the other hand, in CC stem cells, ELK1 is decreased and miR-130b-3p prevails. The study also reports that the cytostatic effects of upregulated miR-130b-5p can be reversed byELK1 overexpression while ELK1 silencing can inhibit cell growth both in vitro and in vivo [245]. The expression of ELK1 has also been found to be regulated by the long noncoding RNA TDRG1 [90]. LncRNA TDRG1 was found to sponge miR-330-5p and thus induce ELK1 expression in CC [90]. TDRG1 is found upregulated in CC tumors and has pro-oncogenic activity while its downregulation leads to tumor suppression due to miR-330-5p activity that downregulated ELK1-related gene expression [90]. A regulator of ELK1 expression has also been shown to be the micro-RNA miR-143-5p [246]. A sponge of miR-143-5p, the long noncoding RNA TCONS_00026907 has been found to be significantly upregulated in CC tissues and cells and also be a poor prognosis factor [246]. The study by Jin et al. in 2017 proposed lnc-RNA TCONS_00026907 as an oncogene that acts by inhibiting miR-143-5p and thus changing the expression of ELK1 among other TFs [246]. Silencing leads to tumor suppression of in vivo models with a pattern similar to ELK1 silencing as reported in the same study [246].

**Table 5:** ELK1 targeting micro-RNAs (miRs) in cancer.

Cancer	microRNA	Regulation by ELK1	Mechanism of Action	Outcome	Ref.
NSCLC	miR-30c	Upregulation	Targets tumor suppressor genes such as *NF1*, *RASA1*, *BID*, *RASSF8*	Drug resistance, cell migration and invasion	[35]
NSCLC	miR-21	Upregulation	Targets tumor suppressor genes such as *NF1*, *RASA1*, *BID*, *RASSF8*	Drug resistance, cell migration and invasion	[35]
BC	miR-200b	Downregulation	miR-200b, upon activation regulates the activity of both ETV4 and ELK1 through regulation of the PIN1-ERK1/2 pathway	Promotion of cancer cell survival during metastasis	[85]
CRC	miR-31-5p	Upregulation	Targets the *CDIP* gene expression	Promotion of metastasis and regulation of autophagy, and apoptosis	[123]
CRC	miR-181a-5p	Upregulation	Targets the *SOCS3* gene expression	Promotion of TME remodeling	[126]
CRC	MIR17HG	Upregulation	Targets the miR-138-5p, a micro-RNA that downregulates HK1	Promotion of glycolysis	[128]
CC	miR-130b-5p	Downregulation	ELK1 suppresses miR-130-5p	Promotion of proliferation	[245]
OC	miR-134	Downregulation	ELK1 downregulates the expression of miR-134	Drug resistance, cancer progression	[174]

Abbreviations: NSCLC = Non-small cell lung cancer; BC = Breast cancer; CRC = Colorectal cancer; CC = Cervical cancer; OC = Ovarian cancer.

Besides RNA-related gene expression, ELK1 has also been investigated as a downstream component of the RAS-RAF-MEK-MAPK pathway, since inhibition of ERK1/2 signaling is a common mechanistic approach of several therapies. A significant CC risk factor is infection with Human papillomavirus 18 (HPV-18), which is theorized to aid tumorigenesis via the E7 oncoprotein [247]. A recent study by Go et al. in 2022 reports that E7 binds to ELK1, increasing its activity [247]. The study also identified targeted genes including *EGR1*, *FOS*, and E2F family of transcription factors, all being known CC risk factors [247]. Moreover, ELK1 was identified as the TF recruited in the promoter regions of the H2A.Z variant histone 1 (*H2AZ1*) and H2A.Z variant histone 1 (*H2AZ2*), both of which were found overexpressed in CC [248]. Both H2A.Z.1 and H2A.Z.2 are credited with pro-oncogenic roles in CC, mainly through assisting the recruiting of TFs in the promoters of genes associated with cancer progression and specifically cell proliferation like NRF1, Nuclear transcription factor Y subunit alpha (NFYA), and RNA Polymerase II (RNA Pol II) [248]. In a study by Pallai et al. in 2012, ELK1 and ETS1 were identified as inducers of the transcription of the known oncogene *CIP2A*, in endometrial, liver, and cervical cancer cells [235]. CIP2A assists MYC’s stabilization, thus promoting proliferation, migration, and invasion [51,235]. Pro-oncogenic activity of the CagA protein found on the *Helicobacter pylori* was firstly identified on HeLa cells [181]. Although *H. pylori* infections are mostly discussed in the context of GC, the study underscores how protein domains found on CagA can affect signal transduction of the gastric mucosa and given the similarity of the two tissues (stomach and cervix), it could provide significant mechanistic insight about CC as well. In another study, investigating whether Caveolin-2 (CAV2) expression modulation could have a therapeutic interest, it was found that CAV2 expression has differential outcomes in each cancer type [234]. In CC, CAV2 leads to elevated cell proliferation while its expression has been found to decrease the activity of STAT3 and ELK1 [234]. Another study showed that phospholipase A and acyltransferase 3 (PLAAT3) (also known as HRASLS3, H-rev107, or PLA2G16) exhibits phospholipase A/acyltransferase (PLA/AT) activity and downregulates H-Ras expression, also acts by ELK1 downregulation [249]. These findings support PLAAT3 as a silencer of ELK1 signaling via Ras deactivation, underscoring how proteins with PLA/AT activity could effectively reduce oncogenic MAPK-ELK1 signal transduction [249]. Regarding tumor suppressor activity, *RIGI* has also been identified as a gene regulating ELK1’s expression in CC. RIG-I overexpression was found to downregulate ELK1, c-Jun, and DDIT3 (CHOP) in both GC and CC cells.

Several substances have also been shown to act by targeting ELK1 expression or activation. The flavonoid Luteolin was shown to decrease the expression of several pro-survival genes including *ELK1*, *MAPK14*, *MAP3K5*, *MAPK3* and *MAPK1*, indicating the suppression of the RAS-RAF-MEK-MAPK signaling pathway [197]. The extract of *Kaempferia parviflora* (commonly known as Thai black ginger) has been shown to inhibit MAPK and PI3K-Akt signaling, leading to significant reduction in PI3K, AKT, ERK1/2, and ELK1 phosphorylation levels [198]. These inhibitory effects also translate into augmented cytotoxicity via apoptosis induction, highlighting ELK1’s phosphorylation in sustaining CC cell viability [198]. A study by Dun and Gao in 2019 demonstrated a negative correlation between Tanshinone I and ELK1 [199]. The first is known for its counter-metastatic and counter-chemoresistance roles in CC while ELK1 is a well-known pro-oncogenic TF [199]. The study reports that ELK1 can promote KRAS synthesis by binding to the gene’s promoter and positively regulate its expression [199]. Tanshinone I effectively downregulates KRAS expression by interfering with ELK1; whereas, artificial overexpression of both KRAS or ELK1 is able to reverse cisplatin-resensitization (achieved by Tanshinone I treatment), thus rendering the cells resistant [199].

### 2.10. Bladder Cancer

**Figure 3:**
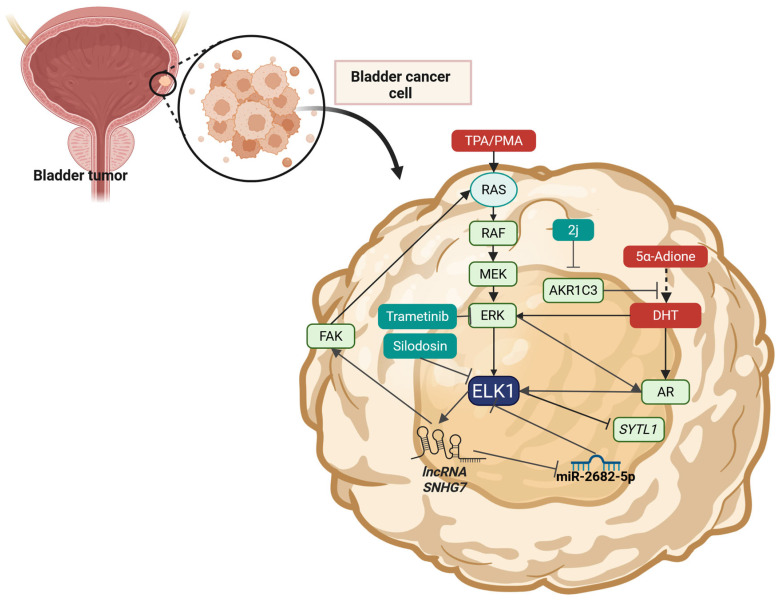
ELK1 in Bladder Cancer. This figure summarizes key pathways in which ELK1 is implicated in Bladder cancer (BCa). Pointy solid-line arrows stand for direct activation while arrows with fading-tails represent indirect activation. Blunt solid-line arrows stand for direct inhibition while blunt arrows with fading tails stand for indirect inhibition. Carcinogenic/oncogenic substances are depicted red and anticancer agents light blue. Created in BioRender. Kalampounias, G. (2025) https://BioRender.com/22zro29.

ELK1 was reported for the first time in Bladder cancer (BCa) cell lines in 2003 [250]. ELK1 activation was reported as a downstream target of MAPK signaling and the authors mentioned that MEK, ERK, and ELK1 phosphorylation was more intense in normal compared to cancerous cell lines at baseline conditions [250]. The authors reported that incubation with 12-O-tetra-decanoylphorbol 13-acetate (TPA) which is a Protein kinase C (PKC) agonist, leads to profound ELK1 activation in urinary bladder transitional cell carcinoma (TCC) cell lines [250] (Figure 3). In the same BCa subtype, TCC, a study revealed that ELK1 expression is elevated and that its expression is not correlated to PKCα expression [251].

In later studies, synergy between ELK1 and the Androgen receptor (AR) were extensively investigated. In 2015, Kawahara et al. documented that stimulation of AR-positive BCa cells with androgen led to significant ELK1 expression and phosphorylation [252]. Androgen was found to promote the nuclear translocation of ELK1 and to increase the proliferation, migration, and invasion rate [252]. On the other hand, ELK1 suppression in urothelial cells lines led to a decline is cell proliferation ability, indicating its significant role in pro-survival and pro-proliferation signal transduction [252]. The authors suggested that ELK1 signaling requires AR activation in order to affect proliferation but not migratory abilities [252]. Migration was shown to be independent of ELK1-AR interactions [252]. Finally, the study reports that ELK1 is required in order for androgen to increase AR transcription activity, underscoring the significant interaction between the two proteins [252]. In another study by Kawahara et al., ELK1 dephosphorylation by Silodosin was found to increase cisplatin toxicity in BCa cells (Table 6) [175]. Silodosin was found to impair the growth of androgen-deprived cells in a manner similar to ELK1 silencing, especially in AR-positive cells, since the two pathways seems to synergize [175]. Finally, he authors even correlated the activation of ELK1 (p-ELK1) to resistance against cisplatin and other chemotherapeutics [175]. The same group also studied whether ELK1 can promote the transformation of normal urothelial cells into neoplastic BCa cells [253]. ELK1 was found to be highly expressed in AR-positive cells, compared to their AR-negative counterparts [253]. The AR-positive cells were found to be more susceptible to tumorigenesis following treatment with 3-methylcholanthralene via ELK1 activation, while Silodosin was accredited with cytoprotective activity by deactivating ELK1 in such cells [253]. Activated ELK1 in upper urinary tract urothelial carcinoma (UUTUC) was also identified as biomarker of poor prognosis [254]. A 2022 clinical trial investigated ELK1 among other potential biomarkers as a distinctive molecular signature of either urothelial bladder cancer (UBC) or upper tract urothelial carcinoma (UTUC) [255]. The authors concluded that ELK1 expression is indeed different between the two cancer subtypes [255].

**Table 6:** Pharmaceuticals targeting ELK1 in lung, bladder, pancreatic, renal, endometrial, and skin cancer.

Cancer	Substance	Model/Sample	Effect(s) on ELK1	Mechanism	Outcome	Ref.
BCa	Silodosin	Patient-derived tissues (from BCa patients) and human urothelial carcinoma cell lines (TCCSUP, UM-UC-3, and 5637)	Decrease in ELK1 expression and phosphorylation	Selectively blocking of α1A-adrenergic receptors and inhibition of RAS-RAF-MEK-MAPK-ELK1 signaling	Reduced viability and migration	[175]
BCa	Trametinib	Dog BCa organoids, mice xenografts	Decrease in ELK1 expression and phosphorylation	Inhibition of ERK1/2-mediated signaling and decrease in the levels of ELK1, MYC, SIK1, and PLA2G4A	Reduced viability	[256]
PaCa	Everolimus	Human PaCa cell lines (Panc-1 and PaCa) and mice xenografts	Bypass of ELK1’s suppressive activity	Inhibition of the PI3K-Akt signaling pathway and surpass the ELK1-imposed suppression of DEPTOR	Reduced viability and resensitization to gemcitabine	[257]
RCC	6-anilino-5,8-quinolinequinone (LY83583)	Human RCC cell line (786-0)	Decrease in ELK1 phosphorylation	Dephosphorylation of ERK1/2, decline of activated ELK1 levels and subsequent downregulation of PTGS2 and BCL2L1.	Apoptosis induction	[258]
EC	Sorafenib	Human EC cell lines (HEC1A, HEC1B, and RL95-2)	Decrease in ELK1 phosphorylation	Dephosphorylation of ERK1/2, decline of activated ELK1 levels and subsequent downregulation of MCL1.	Apoptosis induction	[259]
Melanoma	Mebendazole combined with trametinib	Human melanoma cell lines derived from metastatic patients and, established human melanoma cell lines (BAK, BUL, and STU) and mice xenografts	Decrease in ELK1 phosphorylation	Inhibition of RAS-RAF-MEK-MAPK-ELK1 signaling	Decrease in cell cycle progression, proliferation, and migration	[260]
Melanoma	Fused naphthofuro [3,2-c] quinoline-6,7,12-triones and pyrano [3,2-c]quinoline-6,7,8,13-tetraones derivatives	In vitro study using the NCI-60 panel of tumor cell lines	Decrease in ELK1 phosphorylation	Inhibition of MAPK activation	Apoptosis induction	[261]
Melanoma	Paclitaxel	Human melanoma cell lines (A375 and BLM)	Increase in ELK1 phosphorylation	Persistent RAS-RAF-MEK-MAPK pathway activation	Apoptosis induction	[262]
Melanoma	Carvedilol	Human skin cancer (JB6 Cl 41-5a), human melanoma cell line (A375), and mice xenografts	Reversal of EGF-induced activation	ERK1/2 are phosphorylated in the cytoplasm and do not translocate to the nucleus	Melanoma prevention	[263,264]

Abbreviations: BCa = Bladder cancer; PaCa = Pancreatic cancer; RCC = Renal cell carcinoma; EC = Endometrial cancer.

More recently, a 2021 study by Elbadawy et al. recognized ELK1 gene transcription as a target of trametinib therapy using canine BCa organoids [256]. Trametinib was found to inhibit ERK1/2 mediated signaling and decrease the levels of the ELK1, MYC, Salt-inducible kinase 1 (Serine/threonine-protein kinase, SIK1), and Phospholipase A2 group IVA (PLA2G4A) [256]. A 2022 study identified ELK1 as a regulator of Synaptotagmin-like protein 1 (SYTL1) expression [265]. ELK1 was reported to be upregulated in BCa and its knockdown led to a decrease in cell proliferation and aggressiveness [265]. The study suggests that ELK1 synergizes with Histone deacetylase 2 (HDAC2) and bind to the SYTL1 promoter, suppressing its expression. ELK1 knockdown reverse SYTL1 suppression and leads to tumor suppression [265]. Recently, in a 2024 study, ELK1 was also reported in the context of gemcitabine/cisplatin drug resistance showing similar expression patterns to AR [266]. In this study, by Himura et al., Aldo-keto reductase 1C3 (AKR1C3) is investigated as target in AR-sensitive cancers [266]. Treatment with 5α-Adione which is an AKR1C3 substrate induced both AR and ELK1 expression in drug resistant cells, while the AKR1C3 inhibitor 2j reversed ELK1 and AR overexpression [266]. The study underscores how ELK1 and AR are co-regulated and can be significant contributors in gemcitabine/cisplatin resistance, while their silencing resensitized the cells. Another study implicated ELK1 in a feedback loop mechanism that promotes cell proliferation, migration, and invasion [267]. ELK1 was found to be a regulator of the lncRNA Small nucleolar RNA host gene 7 (SNHG7) which has a known pro-oncogenic role. SNHG7 was found upregulated in BCa and was correlated to poor prognosis [267]. SNHG7 knockdown leads to Proliferating cell nuclear antigen (PCNA) and Ki67 reductions and increases in pro-apoptotic proteins [267]. ELK1 was identified as the transcription activator of SNHG7 synthesis since ELK1 overexpression leads to increased SNHG7 expression while ELK1 silencing diminishes SNHG7 levels [267]. Finally, SNHG7 was identified as sponge of miR-2682-5p micro-RNA which was identified to be negatively correlated to ELK1 and SNHG7 expression [267].

### 2.11. Pancreatic Cancer

#### 2.11.1. Pancreatic Carcinoma

**Figure 4:**
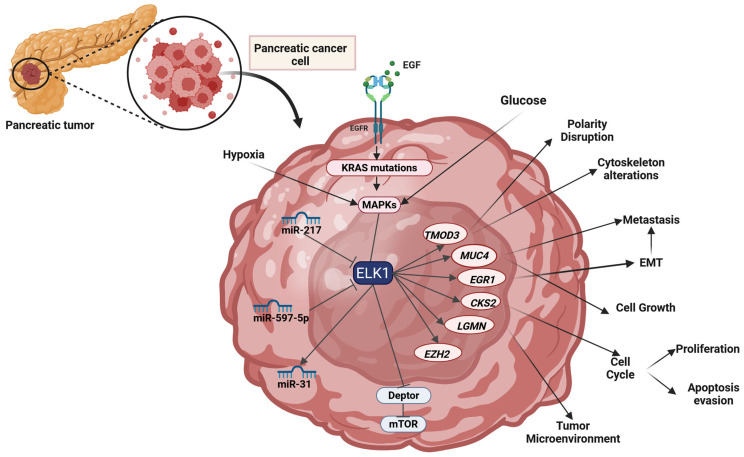
ELK1 in Pancreatic Cancer. This figure summarizes key pathways in which ELK1 is implicated in Pancreatic cancer (PaCa). Pointy solid-line arrows stand for direct activation while arrows with fading-tails represent indirect activation. Blunt solid-line arrows stand for direct inhibition while blunt arrows with fading tails stand for indirect inhibition. Created in BioRender. Kalampounias, G. (2025) https://BioRender.com/qpt874m.

In pancreatic cancer (PaCa), ELK1 has been found to regulate the expression of several genes contributing to tumor growth, invasiveness, metastasis, and chemoresistance. Disrupted RAS-RAF-MEK-MAPK signaling through activating *Ras* mutation has been reported as a major modulator of ELK1 in PaCa [66,268,269,270,271,272]. A mechanism of how the disrupted Ras signaling can promote PaCa was proposed when the relation between EZH2 and ELK1 was explained (Figure 4). EZH2, a histone-modifier protein, can remodel gene expression and make significant changes to the cell. EZH2 was shown to be dependent on ELK1 to facilitate its expression since ELK1 inhibition (or knocking-down) leads to EZH2 downregulation [66]. Activating *KRAS* mutations are a specific subset of MAPK signaling disruptions that lead to tumorigenesis in PaCa and other cancer types. More specifically, PaCa tumors bearing *KRAS* mutations represent the vast majority of diagnosed cases (>95%) [272]. The mutant alleles significantly increase tumor aggressiveness, including metastatic potential and chemoresistance. The promoter of Rho guanine nucleotide exchange factor (*ARHGEF2*), which is essential in tumorigenesis has been shown to be positively regulated by ELK1 [271]. The oncogene *MYC* has also been found to be strongly activated by ELK1, a process which contributes to cell cycle progression and cell growth [273]. Several tumorigenic processes are also partially assisted by cytoskeleton dynamics modifications, mediated by proteins like Tropomodulin-3 (TMOD3). TMOD3 regulates stress fibers and cell polarity and has been found elevated in various cancer types. He et al. in 2025 showed that PaCa cells with mutated *KRAS* alleles had increased TMOD3 expression, which was found to be dependent on ELK1 activation, as a downstream Ras signaling target [272]. Besides the cytoskeleton, ELK1 has also been found to affect tumor microenvironment in PaCa, through modulation of the cysteine protease Legumain (LGMN) [274]. The study by Yan et al. showed that *LGMN* expression is positively correlated with ELK1 and they also proved that ELK1 overexpression in PaCa increases proliferation, invasion both in vitro and in vivo [274]. ELK1 has been found to act as an indirect cell cycle regulator by modulating CDC28 protein kinase regulatory subunit 2 (CKS2) expression. Increased CKS2 has been correlated to poor prognosis, and the gene’s dependency on ELK1 further emphasized the TF’s pivotal role [275]. ELK1 has also been reported as regulator of miR-31 expression, which is a microRNA with contradictory roles in tumorigenesis. KRAS mutations have been found to affect miR-31 expression patterns and the connecting link between disrupted RAS-MAPK signaling and miR-31 transcription was found to be ELK1 [269]. In another study on BxPC-3 cells, ELK1 was found to promote the expression of MUC4, which is a pancreas-specific marker and also has been attributed with roles in cancer cell growth and metastasis [276].

ELK1 targeting has been suggested as a way to suppress PaCa tumor growth and aggressiveness. In drug resistance, the expression levels of ELK1 have been reported elevated [257,277,278]. In hypoxia-induced chemotherapy resistance, several genes have been reported upregulated, among which, ELK1 had the most significant change [278]. Resistance to Genistein has also been connected to ELK1 overexpression; Li et al. in 2021 showed that ELK1 contributes to resistance through the PI3K-AKT-mTOR pathway [257]. ELK1 overexpression suppressed DEP domain-containing mTOR-interacting protein (DEPTOR), which is an mTOR inhibitor, by binding to the proteins promoter [257]. Genistein-resistant cells are thus able to bypass cytotoxicity by maintaining continuously active mTOR signaling [257]. The same study describes how everolimus, an mTOR antagonist can inhibit PI3K-AKT signal transduction and surpass the ELK1-imposed suppression of DEPTOR [257]. Moreover, ELK1 has been identified as a molecular target of miR-217, which can exhibit tumor-suppressing activity in various cancers. Panebianco et al. in 2021 showed that forced expression of miR-217 caused a significant reduction in ELK1 levels and resensitized (previously resistant) cells to gemcitabine [277]. Another study showed that miR-597-5p can directly target and suppress ELK1 levels in PaCa, inducing apoptosis, and leading to tumor growth inhibition [279].

#### 2.11.2. Insulinoma

ELK1 has also been reported as an upregulated transcription factor is a PaCa subtype characterized by insulin overproduction, namely insulinoma. In insulinoma cell lines, activation of ELK1 via MAPK signaling has been found to be a result of B-Raf activation by glucose [271]. B-Raf has been found to increase phosphorylated ELK1, leading in its subsequent activation and transcription of target genes [271]. An identified target of ELK1 activation is *EGR1* gene, which is a known EMT promoter and metastasis driver [280], and the transcription of which is induced [268,281]. ELK1 has also been found to be affected by dinutuximab beta treatment, which leads to a significant decrease in ELK1 levels and induce cell death [282].

### 2.12. Renal Cancer

**Figure 5:**
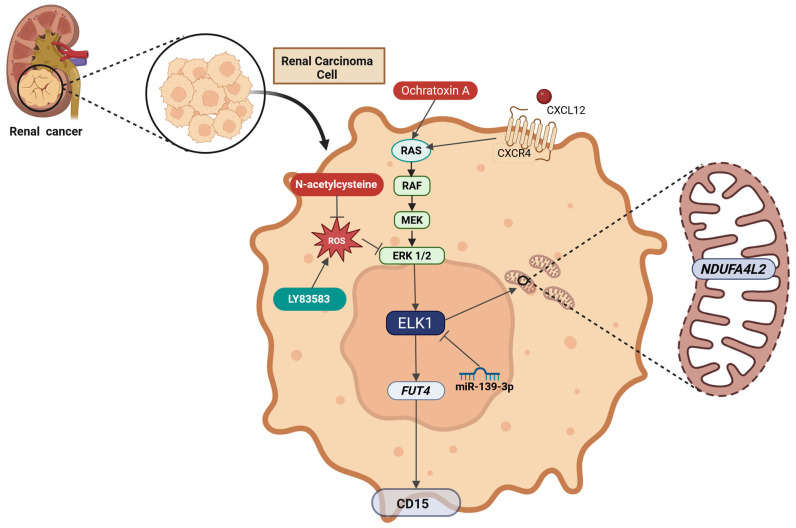
ELK1 in Renal Cancer. This figure summarizes key pathways in which ELK1 is implicated in Renal cell carcinoma (RCC). Pointy solid-line arrows stand for direct activation while arrows with fading-tails represent indirect activation. Blunt solid-line arrows stand for direct inhibition while blunt arrows with fading tails stand for indirect inhibition. Carcinogenic/oncogenic substances are depicted red and anticancer agents light blue. Created in BioRender. Kalampounias, G. (2025) https://BioRender.com/exymo8h.

Regarding renal carcinomas, only limited data exists about the role of ELK1 in tumorigenesis and chemoresistance. A study by Ambrose et al. in 2006 investigated 6-anilino-5,8-quinolinequinone (LY83583) leads to Renal cell carcinoma (RCC) apoptosis [258] (Figure 5). The authors reported ERK1/2 dephosphorylation, which led to a decline in activated ELK1 levels and a subsequent downregulation of PTGS2 and Bcl-2-like protein 1 (BCL2L1; also known as B-cell lymphoma-extra-large, Bcl-XL) [258]. Additionally, increased reactive oxygen species (ROS) formation was documented which the authors reversed by treating the cells the antioxidant N-acetylcysteine (NAC). When NAC was administered, MAPK signaling activity was restored [258], directly connecting redox activity of the cell with ELK1 expression patterns. In a 2007 study employing rat models of renal carcinoma, ELK1 was investigated for a potential role in renal carcinogenicity [283]. The models were fed with ochratoxin A and long-term exposure revealed that ELK1 phosphorylation levels were higher in the treated subjects [283]. Elk1’s activation was attributed to the RAS-RAF-MEK-MAPK pathway [283]. More recently, a study on Clear cell Renal cell carcinoma (ccRCC) revealed that ELK1 is a regulator of NADH dehydrogenase (ubiquinone) 1 alpha subcomplex 4-like 2 (NDUFA4L2) [284]. NDUFA4L2 was found to be upregulated in ccRCC and was correlated to poor prognosis, cancer cell proliferation, and apoptosis evasion [284]. ELK1 was correlated with NDUFA4L2 expression which was verified with ELK1 knockdown experiments [284]. Knockdown of ELK1 in ccRCC cells led to decreased NDUFA4L2 protein levels, further supporting the claims of the authors [284]. Finally, a study on immortalized embryonic kidney cells revealed that the stimulation of C-X-C motif chemokine receptor 4 (CXCR4) by C-X-C motif chemokine ligand 12 (CXCL12), namely the CXCL12-CXCR4 axis, can activate ELK1, which in turn promotes the expression of the Fucosyltransferase 4 (*FUT4*) gene [285]. This process maintains cells in an undifferentiated state, increasing the cell’s oncogenicity potential [285]. A more recent study by Okada et al. (2020), investigated the regulation of the tumor suppressing micro-RNA miR-139 duplex (miR-139-3p/miR-139-5p) in RCC and demonstrated that ELK1 is one of miR-139-3p targets [286]. The study emphasizes that ELK1 is one of the genes with the highest expression in patients with poor prognosis and can serve as an independent prognostic factor for RCC patient survival [286].

### 2.13. Uterine Cancer

Regarding uterine cancer, only a limited number of studies exist on ELK1 and its implications in uterine carcinomas. In 2003, ELK1 was identified as a downstream target of Fibroblast growth factor 7 (FGF7; also known as Keratinocyte growth factor, KGF) and Fibroblast growth factor 10 (FGF10; also known as Keratinocyte growth factor 2, KGF2) in human Endometrial carcinoma (EC) cells [287]. Activation of RAF was reported that led to increases in ELK1 phosphorylation and observable effects regarding cell proliferation [287]. Another study on cervical, endometrial, and liver carcinoma demonstrated that the oncogene CIP2A, is regulated by ELK1 and ETS1 and ultimately leads to augmented cell proliferation [235]. The study revealed that both TFs bind to the CIP2A promoter and that dual inhibition of ELK1 and ETS1 is required to decrease *CIP2A* mRNA levels [235]. The authors also confirmed that in other cell types (human gastric and prostate cancer cells), the same TFs exhibit differential results regarding CIP2A expression, since ETS1 downregulation (without ELK1 silencing) was adequate to decrease CIP2A [51]. In 2013, ELK1 was identified as a target of sorafenib in EC cells [259]. In that study, it is reported that administration of sorafenib inactivates the ERK1/2 pathway, reducing ELK1 phosphorylation levels and thus suppresses MCL1 expression [259]. Finally, in a more recent study (2022), the increased tolerance to ferroptosis of EC cells was attributed to Glutathione peroxidase 4 (GPX4) upregulation, while GPX4 knockdown was found to significantly suppress cell cycle progression, proliferation and migration [288]. On the other hand, apoptosis and ferroptosis were upregulated in GPX4 knockdown cells both in vitro and in vivo [288]. The authors identified ELK1 as the TF regulating GPX4 expression by recognizing ELK1 binding sited on the GPX4 promoter and validated their claims by overexpressing ELK1 which also leads to GPX4 upregulation [288].

### 2.14. Melanoma

**Figure 6:**
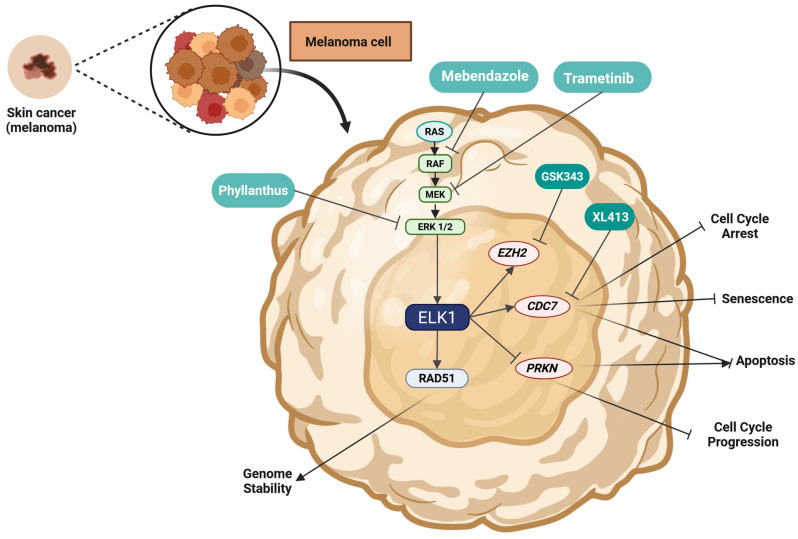
ELK1 in Melanoma. This figure summarizes key pathways in which ELK1 is implicated in Melanoma. Pointy solid-line arrows stand for direct activation while arrows with fading-tails represent indirect activation. Blunt solid-line arrows stand for direct inhibition while blunt arrows with fading tails stand for indirect inhibition. Carcinogenic/oncogenic genes are depicted red and anticancer agents light blue. Created in BioRender. Kalampounias, G. (2025) https://BioRender.com/sb3bncq.

ELK1 has been reported multiple times in melanoma and has been credited with pro-tumorigenic activity in several models. In a 2020 study, ELK1 along with AP-1 and E12 have were credited with the upregulation of key genes in invasive melanoma, correlating its activity with disease progression [289]. The gene targets of ELK1 were found to be upregulated in advanced-stage melanoma, correlating its activity to aggressive disease [289]. Another study focused on Parkin RBR E3 ubiquitin protein ligase (PRKN or PARK2), an E3 ubiquitin ligase with a known tumor-suppressing role and its interaction with ELK1 [290] (Figure 6). In melanoma with BRAF/NRAS mutations, PRKN is downregulated, while BRAF^V600E^ or MAPK inhibition can increase PRKN expression leading to cytostatic and apoptotic effects [290]. The promoter of *PRKN* was identified as an ELK1 target, as ELK1 silencing leads to PRKN overexpression [290]. PRKN has been repeatedly correlated to cell cycle regulation via the proteasomal regulation of cell-cycle regulators. However, ELK1 has also been found to interact with cell cycle progression in an PRKN-independent manner as well. The Cell division cycle 7-related protein kinase (CDC7), a known pro-cell-cycle-progression factor has been found overexpressed in melanoma [291]. Chava et al. in 2022 showed that CDC7 expression is regulated by ELK1, which is also overexpressed in melanoma samples and was shown to promote *CDC7* transcription, leading to enhanced tumor growth and metastatic potential [291]. By interacting with DNA repair protein RAD51 homolog 1 (RAD51 recombinase), a protein crucial in DNA homologous recombination, ELK1 has been found to affect genome stability [292]. RAD51 was found overexpressed in metastatic melanoma cells, supporting DNA damage repair mechanisms and promoting proliferation [292]. RAD51 was found to be a downstream target of RAS-RAF-MEK-MAPK signaling, explaining its prevalences in *RAS-* or *RAF*-mutated tumors [292]. Although several TFs are downstream components of the RAS-RAF-MEK-MAPK pathway, the expression of RAD51 was found to be solely dependent on ELK1’s activation [292]. The study concluded that inhibition of RAD51 can act synergistically with MAPK inhibition (MAPKi), while MAPKi-resistant tumors still response to RAD51 inhibition [292]. Inhibition of ELK1 activation, by targeting the MAPK pathways in melanoma cells had been explored again in previous studies, in which extracts from *Phyllanthus* sp. were found to downregulate the phosphorylation of ELK1 as well as MYC and HIF1A [293]. The anthelminthic agent mebendazole combined with trametinib was also tested on NRAS mutant melanoma cells [260]. Cells with the BRAF^V600K^ and the NRAS^Q61^ mutations were found to be susceptible to dual inhibition by mebendazole and trametinib, which inhibit ELK1 activity [260]. Another study focused on ERK1/2 inhibition in *BRAF*-mutated melanoma, as a way to interfere with the oncogenic Ras signaling. Fused naphthofuro [3,2-c] quinoline-6,7,12-triones and pyrano [3,2-c]quinoline-6,7,8,13-tetraones derivatives were examined as ERK inhibitors and were found to inhibit the phosphorylation of both MAPKs [261]. The effect was evident on the phosphorylation of ELK1 which was reduced, leading to apoptosis [261]. Increased ELK1 activation has also been reported as a consequence of paclitaxel treatment in melanoma cells, due to RAS-RAF-MEK-MAPK pathway activation [262]. The study confirmed that ELK1’s activation was ERK1/2-dependent by administering the ERK1/2 inhibitor PD98059 [262].

Finally, ELK1 has also been assessed in the context of carvedilol-mediated melanoma prevention [263,264]. Carvedilol is one of the few β-blockers able to induce ERK1/2 phosphorylation; however, compared to other RAS-RAF-MEK-MAPK stimulants like EGF, carvedilol does not induce tumorigenesis [294,295]. In this study by Cleveland et al., it was shown that although carvedilol induced the phosphorylation of ERK1/2, activation of ELK1 did not occur [263]. It was also shown that EGF induces ELK1 binding to promoters; however, carvedilol can reverse this. The study concluded that carvedilol phosphorylated ERK1/2 in the cytoplasm and the phosphorylated kinases do not translocate to the nucleus, while during EGF treatment, ERK1/2 are transported in the nucleus and phosphorylation of ELK1 is performed [263]. Since carvedilol exhibits β-blocker activity, potential implementation into cancer-preventive schemes could have serious collateral effects regarding the cardiovascular system [264]. Another study identified R-carvedilol, the enantiomer without β-blocking activity, as a potential mediator of cancer-preventive activity, since it successfully stimulates the RAS-RAF-MEK-MAPK pathway, without leading to ELK1 activation [264].

### 2.15. Ovarian Cancer

In Ovarian cancer (OC), ELK1 was reported for the first time in 2002, as a downstream target of Follicle-stimulating hormone (FSH) signaling [296]. The growth-related effects of FSH signaling were determined to be MAPK-dependent and phosphorylation of ELK1 was reported in neoplastic ovarian surface epithelial cells [296]. ELK1 was reported to be activated by ATP stimulation in a 2003 study investigating ovarian tumorigenesis [297]. Another role of ELK1 in OC tumorigenesis was identified by studying the inflammatory mediator Prostaglandin E2 receptor EP3 subtype (PTGER3) [176]. The study reported that PTGER3 is highly expressed in OC patients who have acquired resistance to cisplatin and its silencing using siRNAs leads to a downregulation of cancer cell proliferation, migration, and invasion rates [176]. Signaling through the RAS-RAF-MEK-MAPK pathway was also reported as affected by PTGER3 [176]. The TFs identified in PTGER3 signaling included ETS1, ELK1, and Cystic fibrosis transmembrane conductance regulator (CFTR or MRP7) while a sustained suppression of PTGER3/ELK1 signaling was shown to resensitize the cells to cisplatin, suggesting the pathway as a potential drug resistance mechanism [176]. A study investigating the hyaluronan-CD44 interactions with IQ motif containing GTPase activating protein 1 (IQGAP1) reported ELK1 activation via the RAS-RAF-MEK-ERK1/2 signaling cascade [298]. Both ELK1 and Estrogen receptor alpha (ERα) were reported as activated which leads to the transcription of genes supporting cell proliferation and migration in OC [298]. Another study focused on the effects of adhesion loss on OC cells [299]. The study demonstrated that following detachment from the matrix, ovarian cancer cells dramatically increase MEK and ERK1/2 signaling while the same phenomenon was not observed in benign cells [299]. ERK activation was documented to increase ELK1 phosphorylation in suspended cells. It was also shown that MEK inhibitors effectively suppressed ERK1/2 activity in non-adherent cells [299]; implying that ERK1/2 and ELK1 activation is crucial for the survival of detached cells. Therefore, the study underscored how ELK1 activation due to loss of adhesion may support metastatic OC cells [299], thus providing a convenient therapeutic target. ELK1’s role in apoptosis evasion was also reported by a 2012 study, in which, ELK1-mediated gene transcription was found to regulate apoptosis via MCL1 overexpression in OC ascites [300]. The inhibition of ERK1/2 signaling or knocking down ELK1 mRNA was found to inhibit the synthesis of MCL1 and leads to induction of apoptosis by the TNF-related apoptosis-inducing (TRAIL) protein [300].

Another role of ELK1 in OC was identified in micro-RNA regulation. ELK1 was found to induce the expression of the lncRNA LBX2-AS1, which in turn sponges the regulatory micro-RNA miR-4784 [41]. The target of miR-4784 was identified to be the Lysine-specific demethylase 5C (*KDM5C*) gene, which is downregulated by the micro-RNA, while LBX2-AS1 overexpression via ELK1 can reverse this [41]. In another study, by Shuang et al. in 2017, ELK1 was found to target miR-134 expression in paclitaxel-resistant OC cells [174]. miR-134 is correlated to tumor-suppressing activity and in drug-resistant cells it is often found downregulated [174]. ELK1, Proto-oncogene c-Rel (REL), and Nuclear factor NF-kappa-B p105 subunit (NFKB1 or p150/p50) were identified as regulators of miR-134 expression and were also reported to be overexpressed in chemo-resistant tumors. miR-134 was found to target the TGF-beta-activated kinase 1 (*TAB1*) gene while its expression was increased following ELK1, REL, and NFKB1 overexpression [174].

In a 2006 study, the mechanism by which the adenovirus type 5 gene *E1A* exhibits suppression of oncogenicity was investigated in OC cell lines [301] (Table 7). *E1A* was found to upregulate the Proliferation and apoptosis adaptor protein 15 (Phosphoprotein Enriched in Astrocytes 15, PEA15), which inhibits the translocation of ERK1/2 inside the nucleus [301]. This suppresses ELK1 activation, since PEA15 knocking down increases nuclear ERK1/2 and also leads to elevated ELK1 levels [301]. Regarding ELK1’s suppression, in a 2011 study 3,3′-Diindolylmethane (DIM) was investigated as a potential EGFR inhibitor, and it was found to decrease ERK1/2-mediated signaling [302]. The activity of MEKs, MAPKs, and ELK1 was found significantly suppressed and xenografts studies also revealed that tumor growth was limited as well, indicting a direct link between ELK1’s deactivation by EGFR blocking and antitumor effects [302]. EGFR and ELK1 have also been identified as the drug targets of the antibiotic monensin in OC cells [303]. Treatment with the antibiotic was found to suppress ELK1 phosphorylation and in general reducing SRF, AP-1, NF-κB, and STAT3 activity and EGFR expression [303]. Monensin was also found to synergize with oxaliplatin and EGFR inhibitors and suppress proliferation and tumor growth using both in vitro an d in vivo models [303]. Besides EGFR signaling, ELK1 in IGF signaling has also been investigated. Deng et al. in 2016 reported that niclosamide, an inhibitor of IGF signaling was found to suppress cell growth and migration by targeting ELK1, AP-1, c-MYC, and NF-κB [304].

**Table 7:** Pharmaceuticals targeting ELK1 in ovarian cancer and gliomas.

Cancer	Substance	Model/Sample	Effect(s) on ELK1	Mechanism	Outcome	Ref.
OC	E1A	Human OC cell lines (SKOV3.ip1 and OVCAR-3)	Decrease in ELK1 phosphorylation	Overexpression of PEA15, and inhibition of the translocation of ERK1/2 inside the nucleus	Tumorgenicity suppression	[301]
OC	3,3′-Diindolylmethane (DIM)	Human OC cell lines (SKOV-3, OVCAR-3, and TOV-21G)	Decrease in ELK1 phosphorylation	Inhibition of EGFR-MAPK-ELK1 signaling	Growth inhibition	[302]
OC	Monensin	Patient-derived OC tissues and human OC cell lines (SKOV3 and HeyA8)	Decrease in ELK1 phosphorylation	Inhibition of EGFR-MAPK-ELK1 signaling and downregulation SRF, AP-1, NF-κB, and STAT3 activity and EGFR expression	Proliferation inhibition, apoptosis induction	[303]
OC	Niclosamide	Human OC cell lines (SKOV3 and HeyA8)	Decrease in ELK1 phosphorylation	Inhibition of IGFR signaling	Reduced cell growth and migration	[304]
Glioma	Amitriptyline	Rat glioma cell line (C6)	Increase in ELK1 phosphorylation	Phosphorylation of ERKs and JNKs	Increase in EGR1 transcription	[305]
GBM	Anisomycin	Human GBM cell line (U-87 MG)	Increase in ELK1 phosphorylation	Phosphorylation of all three major MAPK classes (ERK1/2, JNK, p38)	Increase in EGR1 transcription	[306]
GBM	LY294002 or wortmannin	Human GBM cell line (U-138)	Accumulation of phosphorylated ELK1 in the cytoplasm	AKT inhibition	Growth inhibition	[307]
GBM	UO126	Human GBM cell line (U-138)	Decrease in ELK1 phosphorylation	MEK inhibition	Growth inhibition	[307]
GBM	FR180204	Human GBM cell line (U-138)	Decrease in ELK1 phosphorylation	MAPK inhibition	Growth inhibition	[307]
GBM	Curcumin	Human GBM cell line (U-87MG) and rat glioma cell line (C6)	Increase in ELK1 phosphorylation	Phosphorylation of ERKs and JNKs, activation of EGR1, and upregulation of p21^Waf1/Cip1^	Growth inhibition	[95]

Abbreviations: OC = Ovarian cancer; GBM = Glioblastoma.

Finally, ELK1 was also studied in the context of Gonadotropin-Releasing Hormone II (GnRH-II) antiproliferative effects [308]. GnRH-II treatment reduced cell proliferation and indicated ERK1/2 activation which could be blocked by the PD98059 MAPK inhibitor [308]. ELK1 was reported phosphorylated; however, anti-proliferative actions were attributed to it [308].

### 2.16. Gliomas

#### 2.16.1. Glioma

Several mechanisms of ELK1 activity have been described in gliomas, indicating the TF’s role in multiple aspects of the disease. In 2009, Sahin et al. identified that Elk1, among other ETS family TFs (Ets1, Elf1, Fli1, and Etv1) is overexpressed in C6 rat glioma cells [309]. ELK1 was reported to promote glioma cell proliferation by promoting the expression of the lncRNA PSMB8-AS1 [42]. In Shen et al. (2020), PSMB8-AS1 is reported to be upregulated in gliomas, while its silencing was shown to suppress cell proliferation [42]. PSMB8-AS1 was found to bind to miR-574-5p and this interaction promotes the expression of the pro-proliferation RAS-related protein Rab-10 (RAB10), which is normally targeted by miR-574-5p and thus downregulated [42]. Therefore, an increased ELK1 expression forms an axis that regulates RAB10 expression via silencing of the gene’s main regulator miR-574-5p [42]. A recent study correlated ELK1 activation with the maintenance of telomeres, via Regulator of telomere elongation helicase 1 (RTEL1) [310]. RTLE1 was identified as a proliferation, migration, and invasion promoter both in vitro and in vivo [310]. The expression of RTEL1 was found to be a consequence of ELK1/JNK activation and suggested RTEL1 as a potential marker of prognostic significance [310].

In glioma, ELK1 has also been investigated as a promoter of EGR1 transcription. In a 2007 study on rat C6 glioma cells, Egr1 expression was documented to be stimulated by the tricyclic antidepressant amitriptyline [305]. Amitriptyline was found to induce Elk1 binding to the *Egr1* promoter, following activation of the ERK1/2 and JNK MAPKs, while it is also described how MAPK inhibition suppresses *Egr1* transcription [305]. Chung et al. concludes that the discovered mechanism could shed light on how the antidepressant drugs may exhibit their therapeutic effects [305]; however, it is also important to mention that since this study was performed on glioma cells, its importance may fall beyond antidepressant activity of amitriptyline. Recent studies have investigated the role of tricyclic antidepressants as anticancer agents and amitriptyline has shown such potential [311,312,313,314,315,316,317,318]. Therefore, interactions of amitriptyline with ELK1 should be further studied to better explain its anticancer potential. Regarding ELK1-EGR1 interaction, several alternative mechanisms have been described or proposed by other groups in different types of cancer [47,177,210,259,281], including glioma/glioblastoma [305,306]. Nonetheless, according to Kim et al. (2011), Estrogen receptor beta, (ERβ) can stimulate EGR1 transcription via the Raf-MEK1-MAPK-ELK1 pathway in a non-genomic manner [319].

Targeting of ELK1-mediated gene transcription is a concept investigated not only in glioma but in several tumors. Regarding glioma, a substance isolated from the fruitbody of *Trogia venenata* (commonly known as little white mushroom) named Phragmunis A, was found to exhibit cytotoxicity on glioma cells via targeting of the ELK1/SRF complex [320]. Phragmunis A suppressed the binding efficiency of ELK1 and led to decreased gene expression [320]. Huang et al. also suggested that ELK1’s deactivation leads to suppression of the antiapoptotic MCL1 protein levels, as well as the downregulation of EGR1-BMI1 and the upregulation of F-box/WD repeat-containing protein 7 (FBXW7), thus leading to apoptotic death [320]. Another interaction that has been documented in glioma is that of ELK1 with CAV2 [234]. As mentioned in HCC and CC, CAV2 targeting results in differential results depending on the cancer type [234]. Lee et al. in 2011 reported that Cav2 knockdown in C6 rat glioma cells reduce cell proliferation rate [234]. These findings are of interest since in glioma cells with a downregulated Cav2, the TFs Elk1 and Stat3 are also found downregulated [234], indicating a direct link between Cav2’s oncogenic role and Elk1’s expression patterns.

A very different aspect of ELK1’s role in glioma was investigated by Li et al. in 2018 [321]. In this study, the role of modulators, of proteins that modify how transcription factors act, was investigated regarding ELK1 in brain Lower grade glioma (LGG) [321]. As a key modulating mechanism, the study focused on alternative splicing and concluded that Amyloid precursor protein (APP) and Serine/threonine kinase 16 (STK16) can modulate ELK1’s phosphorylation (and thus enhance its transcriptional activity), based on changes in the exon inclusion ratio of the two modulators [321]. The study concluded that differential alternative splicing of Amyloid beta A4 protein (APP)/Serine/threonine-protein kinase 16 (STK16) is associated with ELK1’s diverse roles in gene transcription and may be of great interest in the context of ELK1 implications in pathophysiology of diseases like glioma [321].

#### 2.16.2. Glioblastoma

ELK1 has also been reported in the most aggressive glioma subtype, namely glioblastoma (GBM). Even though GBM is not a subtype of glioma, its progression rate, treatment approach, and underlying pathophysiological mechanisms, render it substantially different from other gliomas and thus it is analyzed on its own [322,323,324].

EGFR activation of ELK1 in GBM cells has been reported multiple times [325,326]. Shin et al. in 2006 concluded that treatment of human GBM cells (U-87 MG) with the translation inhibitor Anisomycin increases EGR1 accumulation [306]. The mechanism they proposed included activation of ELK1 by the MAPK signaling cascade and a subsequent SRE-mediated EGR1 transcription [306]. The study reports that anisomycin induced the phosphorylation of all three major MAPK classes (ERK1/2, JNK, p38), leading to EGR1 transcription, while treatment with MAPK or MEK inhibitors suppresses EGR1 synthesis [306]. A 2012 study by Mut et al., concluded that EGFR stimulation by EGF leads to ELK1 activation in GBM cells by the RAS-RAF-MEK-MAPK pathway, but that the PI3K-AKT pathway also contributes to ELK1 activation [307]. ELK1 phosphorylation levels were affected inly by inhibition of MEK1 (UO126) or ERK2 (FR180204) while AKT inhibition did not affect its phosphorylation levels [307]. However, the study reports that AKT inhibition (LY294002 or wortmannin) did affect the localization of phosphorylated ELK1 which accumulated in the cytoplasm [307]. Inhibition of ELK1 activity had also been mentioned in Human bronchial epithelial (HBE) cells [327]; however, this was the first mention of ELK1 inhibition by PI3K-AKT inhibition in gliomas/glioblastomas. In a 2015 study by Wang et al., the hematopoietic factor GATA2 was reported as upregulated in GBM and is a marker of poor prognosis [328]. The study investigated whether GATA2 expression can affect cell proliferation, migration, and invasion and it was documented that its silencing led to significant suppression of these cell functions [328]. EFGR and MAPK activation were also found to upregulate the expression of GATA2, which in turn upregulated ELK1 [328]. Given the fact that ELK1 is a downstream molecule of the RAS-RAF-MEK-MAPK pathway, these interactions seem to form a positive feedback loop that propagates MAPK-related signaling and ELK1-realted gene transcription; both of high pro-tumorigenic potential.

EGFR signaling and ELK1 activation were also reported in GBM regarding metabolism regulation [329]. Yang et al. reported in 2020 that glutamine metabolism gets upregulated following EGFR stimulation, via the RAS-RAF-MEK-MAPK pathway that activates ELK1 [329]. ELK1 activation was linked to the transcription of Glutamate dehydrogenase 1 (GDH1), an enzyme which promotes glutamine metabolism [329]. The study also reports that under ELK1-silencing conditions, EGFR stimulation does not lead to glutamine lysis, underscoring the pivotal role of ELK1 in the process [329].

In GBM, another study revealed that an isoform of the protein kinase family, Protein kinase C eta type (PKCη or PRKCH), contributes to tumor progression via activation of ELK1 [330]. Treatment with Phorbol-12-myristate-13-acetate (PMA) which is a known PKC inducer caused significant increases in cell proliferation of human glioblastoma cell lines in a PRKCH-dependent manner [330]. The study revealed that the downstream targets of PRKCH activation were ERK1/2 and ELK1 since a pre-treatment with PKC or ERK1/2 inhibitors halted ELK1-mediated transcription and the expected proliferation [330]. Although the study underscored ELK1’s significance, no target genes were identified following PKC-eta activation [330].

Regarding GBM targeting, a study investigated how curcumin can affect cell growth and came up with an interesting role for ELK1 [95]. Curcumin was found to upregulate the cell cycle regulator p21^Waf1/Cip1^ though activation by EGR1 in human GBM cells [95]. Curcumin was shown to activate EGR1-mediated gene transcription by stimulating the ERK/JNK-ELK1 pathway which activates ELK1 [95]

### 2.17. Osteosarcoma

**Figure 7:**
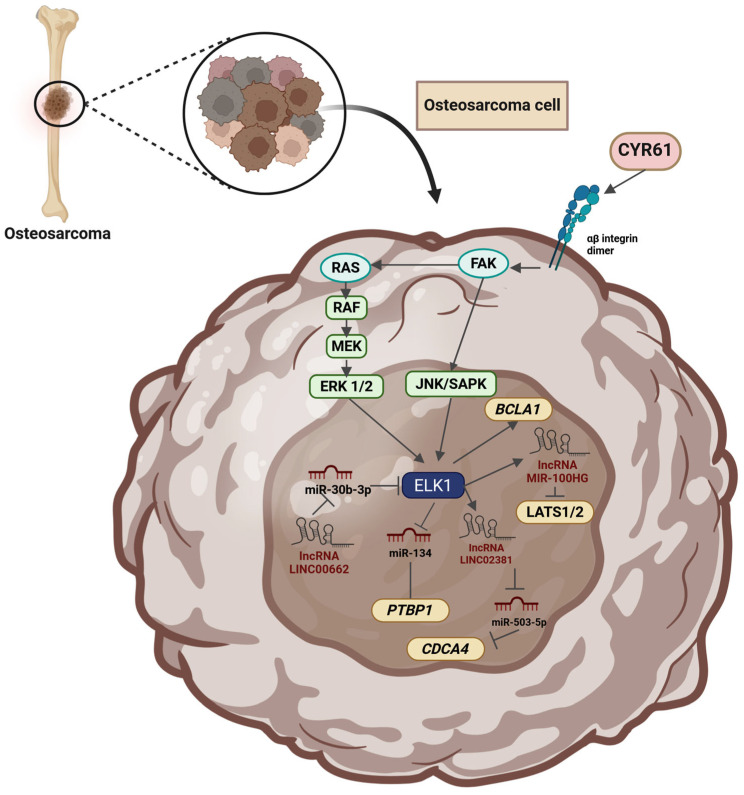
ELK1 in Osteosarcoma. This figure summarizes key pathways in which ELK1 is implicated in Osteosarcoma. Pointy solid-line arrows stand for direct activation while arrows with fading-tails represent indirect activation. Blunt solid-line arrows stand for direct inhibition while blunt arrows with fading tails stand for indirect inhibition. Carcinogenic/oncogenic substances are depicted red. Created in BioRender. Kalampounias, G. (2025) https://BioRender.com/195qp9l.

In osteosarcoma, ELK1 was identified in 2003 as a key contributor in immediate-early gene induction by anisomycin and arsenite [331]. The cellular stress provoked by the two agents increased MAPK signaling since ERK1/2, Stress-Activated Protein Kinase (SAPK/JNK), and p38 were reported phosphorylated and increases in the phosphorylation of CREB, SRF, and ELK1 were reported [331]. ELK1 phosphorylation was found to be dependent on both ERK and JNK, as inhibition of either MAPK class, reduced ELK1 activity; whereas, inhibition of p38 did not affect ELK1 activation [331]. Another implication of ELK1 in osteosarcoma EMT and metastasis was proposed in a 2014 study by Hou et al. [332]. ELK1 was found to promote the expression of EMT markers and cell migration, through activation by Cysteine-rich angiogenic inducer 61 (CYR61; also known as Cellular communication network factor 1, CCN1), via the ERK1/2 pathway [332] (Figure 7). Knockdown of ELK1 was shown to inhibit CYR61-induced migration while treatment with CYR61 was found to promote the activation of RAF, the translocation of ERK1/2 to the nucleus, and ultimately the phosphorylation of ELK1 [332]. ELK1 activation was also reported to control anti-apoptotic Bcl-2-related protein A1 (BCL2A1) expression in osteosarcoma cells, in a 2014 study [333]. Knocking-down ELK1 led to decreases in BCL2A1 expression, while suppression of ERK1/2 signaling had analogous effects [333].

Several recent studies in osteosarcoma focus on how ELK1 regulates the activity of RNA molecules, emphasizing its role as a pro-oncogenic gene silencer [43,44,334,335]. In a 2019 study on osteosarcoma cell lines, ELK1 was found to upregulate the oncogenic lncRNA MIR100HG [43], a finding also reported in gastric cancer by another group [39]. High MIR100HG expression was determined as a poor prognosis factor in osteosarcoma patients, and its upregulation was found to suppress Hippo signaling activity via the epigenetic silencing of Large tumor suppressor kinases 1 and 2 (LATS1/2) [43]. ELK1 was directly correlated as an MIR100HG activator, by directly binding to its promoter; whereas, ELK1 knockdown was found to diminish MIR100HG levels [43]. A similar mechanism was described by Bian et al. in 2021, in which, ELK1 was reported to upregulate lncRNA LINC02381 [44]. LINC02381’s expression was found to be increased in osteosarcoma cell lines and tissues and was identified as a marker of poor prognosis [44]. ELK1 activation was found to be necessary for LINC02381 expression, and it was accredited the role of a miR-503-5p sponge [44]. miR-503-5p was identified as a suppressor of the pro-proliferation Cell division cycle-associated protein 4 (*CDCA4*), which in cases of LINC0283 overexpression is upregulated, due to miR-503-5p sponging [44]. Therefore, an axis connecting ELK1 with CDCA4 was identified, as well as a suppression mechanism of miR-503-5p. Another study reports that the lncRNA LINC00662 is highly expressed in osteosarcoma cells while its suppression leads to proliferation, migration, and invasion activity reduction [334]. The study identified LINC00662 as sponge for the tumor suppressor micro RNA miR-30b-3p, the overexpression of which has anti-proliferative and anti-migratory effects [334]. Finally, a direct link between miR-30b-3p and ELK1 was identified, proposing a new regulatory axis that involves LINC00662/miR-30b-3p/ELK1 with a pro-tumorigenic role in osteosarcoma progression [334]. Finally, ELK1 was identified with a significant role in anthracycline resistance by reversing the silencing of the Polypyrimidine tract-binding protein 1 (*PTBP1*) gene [335]. PTBP1 has a prominent role in the Warburg effect and its activity was found to be a significant contributor of doxorubicin resistance [335]. Resistant in osteosarcoma cells [335]. The study identified miR-134 as a suppressor of PTBP1 gene and ELK1 as a suppressor of miR-134 expression, by binding strongly to its promoter and inhibit transcription [335].

## 3. Conclusions

**Figure 8:**
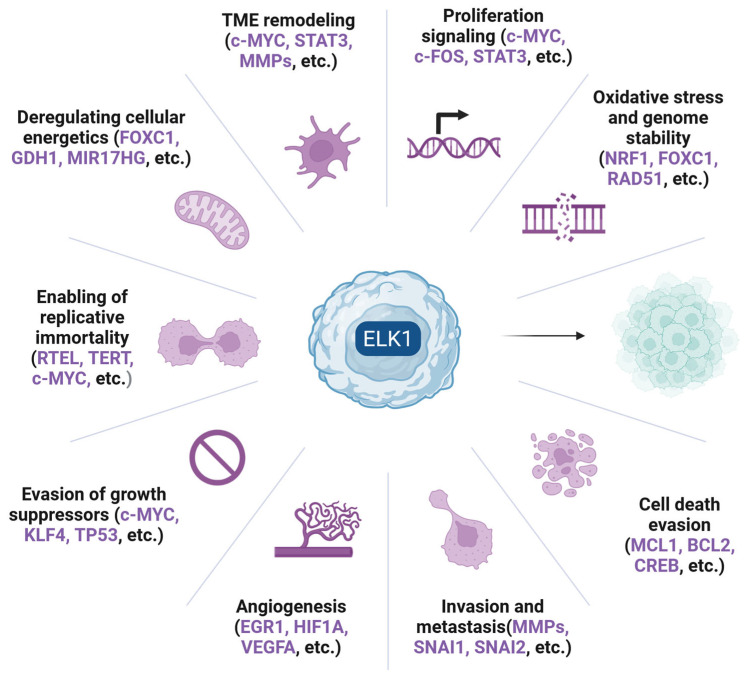
The implications of ELK1 in tumorigenesis and examples of its key downstream targets or upstream regulators of pan-cancer significance. Created in BioRender. Kalampounias, G. (2025) https://BioRender.com/iqv1y4f.

Research data from the last three decades suggest that ELK1 is a pivotal molecule in several solid tumors, underscoring its potential role as a diagnostic or prognostic marker and as a candidate therapeutic target. The extensive literature research presented in this study revealed that ELK1 is a key transcription factor implicated in several cancer hallmarks, including the sustaining of cell proliferation signaling, the evasion of apoptosis and other cytostatic mechanisms, the activation of invasion and metastasis pathways, the epigenetic reprograming of the cancer cells, the dysregulation of the metabolism, the promotion of angiogenesis, and the remodeling of the tumor microenvironment (Figure 8). The vast majority of the studies report that ELK1 is found upregulated in most malignancies (compared to normal tissues) and this upregulation is directly correlated to disease progression. Furthermore, studies with forced overexpression of ELK1 confirmed that it can exert oncogenic potential, while conversely, several studies on ELK1-knockdown cancer cells report that its suppression or deletion effectively downregulates cell growth and promotes apoptosis. Therefore, both aspects emphasize that its targeting could have therapeutic applications. Nonetheless, these studies are based on cancerous cells in which ELK1 is activated (via the aforementioned mechanisms) and is an established part of the tumor-supporting machinery. It is noteworthy that ELK1’s “natural state” is a mostly inactivated one, and studies in transgenic mice (not in the context of cancer) have shown that its deletion does not severely affect embryonic development or adult life [336,337]. These studies reported mild impairments of the gene activation in the nervous system and decreased glucose tolerance, indicating that ELK1 is needed for these processes [336,337]. However, other TFs were suspected to (at least) partially compensate for its absence [336]. Since there is a considerable lack of data about these compensatory mechanisms, more research is warranted in this direction as these TFs could be of great interest in the context of carcinogenesis prevention, prognosis, and treatment. ELK1 as an independent prognostic factor has been suggested by numerous researchers, highlighting the protein’s role in tumorigenesis-related processes and its correlation to aggressive form of cancer. According to most data, ELK1 is simultaneously implicated in several functions, acting as a main downstream target of ERK1/2, which are two of the most significant kinases in cancer biology and epicenter of several anticancer approaches.

ELK1 is an integration point for several signaling pathways participating in extensive crosstalk between the RAS-RAF-MEK-ERK pathway, the activation of the stress-related MAPKs (JNKs and p38), the PI3K-AKT pathways, Wnt/β-catenin signaling, Hippo signaling, and cytokine signaling via regulation of the JAK/STAT pathway. Several studies have targeted ELK1 indirectly, by blocking the upstream signal transduction. It is noteworthy that the observed outcome of ELK1’s activation is a combination of its upstream regulation and the tissue- or cancer-specific expressed/activated transcription factors which collaborate with ELK1 to facilitate transcription. Activation of the well-established RAS-RAF-MEK-ERK1/2 pathway occurs as part of early response mechanisms and is mostly correlated to pro-survival activity. Targeting of this pathway is part of several existing therapies and novel approaches are still under development, since MAPK signaling modulation affects multiple cellular functions and it is a convenient target for cancer therapy. Although the MAPK pathway seems to be a major part of this approach, other significant targets are growth-factor-related signaling molecules. Inhibition of EGF and IGF signaling with TKIs was found to significantly impair ELK1-mediated gene transcription, while other experimental substances or plant extracts have shown comparable effectiveness. EGFR- and IGFR-mediated signal transduction lead to activation of ERK1/2 by MEKs (interplay with other pathways is also a significant contributor); however, an important parameter to exhibit pleiotropic activity is the translocation of the phosphorylated ERKs inside the nucleus. The inhibition of this process via blocking the importins was reported to suppress ELK1’s activation and was found to be the mechanism underlying carvedilol’s melanoma-preventive activity, thus underscoring how this notion could be expanded, and more research should be conducted in this direction. Although the vast majority of the studies credit the activation of ELK1 by ERK1/2 with a pro-tumorigenic role, it is important to mention that the activation of ELK1 by the other two MAPK classes (JNKs and p38 MAPKs) has been reported to exhibit differential results ranging from proliferative to apoptotic activity. Moreover, several drugs were reported to act by inducing persistent MAPK activation leading to it has also been implicated in cell-death-related mechanisms, mainly through the transcription of death receptors and pro-apoptotic factors.

Phosphorylated ELK1 synergizes with other TFs to facilitate transcription, and these complexes are of increased significance in tumorigenesis and tumor growth in several cancer types. The interactions of ELK1 with MZF1, AR, and ER are some of the most reported in several cancer types, all credited with growth-related phenomena. The disruption of these complexes has been found to attenuate the effects of ELK1, regardless of its activation by upstream signaling, thus suggesting novel pharmaceutical approaches. Targeting the recruitment of these complexes on gene promoters may be a promising therapy in several cancer types, since upon activation, ELK1 promotes the expression of several oncogenes including *EGR1* and *FOS*. ELK1 has been found to be a component of several chemoresistance pathways, reducing chemosensitivity to cisplatin/oxaliplatin, contributing to resistance against gemcitabine and proteasome inhibitors, and regulating susceptibility to paclitaxel and other pharmaceuticals. Therefore, its effective silencing, inhibition, or targeting of its interactions could provide significant insight into drug resistance. A major part of this concept could be the exploitation of naturally evolved ELK1 regulation mechanisms which also regulate other TFs and could be weaponized against cancer. ELK1 has been reported to be the target of several micro-RNAs, many of them are well characterized as tumor-suppressors (Table 8).

**Table 8:** Micro-RNAs (miRs) targeting ELK1 in cancer.

Cancer	RNA	Effect(s) on ELK1	Mechanism	Outcome	Ref.
BC	miR-135a	Downregulation	Reduction in ELK1/3 levels	Inhibition of proliferation	[86]
BC	miR-326	Downregulation	Reduction in ELK1 levels	Inhibition of proliferation, colony formation, and invasion	[87,89]
BC	miR-330-5p	Downregulation	Reduction in ELK1 levels	Inhibition of proliferation and migration, and induction of apoptosis	[89]
CRC	miR-206	Indirect downregulation	Downregulation of the Met/ERK/ELK1/HIF-1α/VEGF-A pathway	Angiogenesis inhibition	[111]
CRC	miR-873	Downregulation	Binding to the 3′UTR of *ELK1* and *STRN4* mRNAs, inhibiting their translation	Inhibition of proliferation and migration	[124]
CRC	miR-382-5p	Downregulation	Reduction in ELK1 levels	Limits cancer progression	[125]
PCa	miRNA let-7a	Indirect downregulation	Downregulation of IGF1R’s expression and thus decreased ELK1 activation and c-FOS expression	Limits cancer progression	[161]
GC	miR-139-3p	Downregulation	Reduction in ELK1 levels	Limits GC tumorigenesis	[190]
ESCA	miR-29a-3p	Downregulation	Reduction in ELK1 levels	Inhibition of proliferation, migration, and invasion	[202]
LSCC	miR-340-3p	Downregulation	Reduction in ELK1 levels	Inhibition of proliferation, migration, colony formation, and invasion	[208]
HCC	miR-361-3p	Downregulation	Reduction in ELK1 levels	Inhibition of proliferation, migration, and invasion	[21]
CC	miR-197-3p	Downregulation	Reduction in ELK1 levels	Inhibition of cell cycle progression, reduction in viability, induction of apoptosis and autophagy	[243]
CC	miR-326	Downregulation	Reduction in ELK1 levels	Inhibition of proliferation, colony formation, and invasion	[88]
CC	miR-130b-5p	Downregulation	Overexpression of miR-130b-5p leads to reduction in ELK1 levels	Inhibition of proliferation	[245]
CC	miR-330-5p	Downregulation	Reduction in ELK1 levels	Downregulation of ELK1-related gene expression	[90]
CC	miR-143-5p	Downregulation	Reduction in ELK1 levels	Limits cancer progression	[246]
BCa	miR-2682-5p	Downregulation	Reduction in ELK1 and lncRNA SNHG7 levels	Inhibition of cell proliferation, migration, and invasion	[267]
PaCa	miR-217	Downregulation	Reduction in ELK1 levels	Limits cancer progression and resensitizes cells to gemcitabine	[277]
PaCa	miR-597-5p	Downregulation	Reduction in ELK1 levels	Induction of apoptosis, and inhibition of tumor growth	[279]
RCC	miR-139-3p	Downregulation	Reduction in ELK1 levels	Limits cancer progression	[286]
Osteosarcoma	miR-30b-3p	Downregulation	Binding to the 3′UTR of *ELK1* and downregulation of its transcription	Inhibition of proliferation, migration, and invasion	[334]

Abbreviations. BC = Breast cancer; CRC = Colorectal cancer; PCa = Prostate cancer; GC = Gastric cancer; ESCA = Esophageal cancer; LSCC = Laryngeal squamous cell carcinoma; HCC = Hepatocellular carcinoma; CC = Cervical cancer; BCa = Bladder cancer; PaCa = Pancreatic cancer; RCC = Renal cell carcinoma.

Micro-RNAs like miR-326, miR-330-5p and others have been reported to target the 3′UTR of *ELK1* mRNA and thus downregulate its expression. These miRs are often reported downregulated in advanced disease stages and their expression was negatively correlated with that of ELK1’s. Even though there is limited data on whether some of these miRs indeed regulate ELK1 in all cancer types or whether each described mechanism is cancer-type-specific, the significance of this mechanism is great as these could be part of future therapeutic approaches. Besides being regulated by noncoding RNAs at the post-transcriptional levels, ELK1 regulates the expression of miRs and lncRNAs with known pro- or anti-tumorigenic activity at the transcriptional level. Several closed feedback loops have been reported, involving micro-, circular- and long-noncoding-RNAs, the dysregulation of which could become the target of future therapies.

## Data Availability

No new data were created or analyzed in this study.
